# Changes in Online Food Access During the COVID-19 Pandemic and Associations With Deprivation: Longitudinal Analysis

**DOI:** 10.2196/41822

**Published:** 2023-04-17

**Authors:** Matthew Keeble, Jean Adams, Thomas Burgoine

**Affiliations:** 1 MRC Epidemiology Unit, Institute of Metabolic Science University of Cambridge School of Clinical Medicine Cambridge United Kingdom

**Keywords:** COVID-19, digital food environment, fast foods, online food delivery services, public health

## Abstract

**Background:**

Food prepared out of home is typically energy-dense and nutrient-poor. Online food delivery services have become a popular way to purchase such food. The number of accessible food outlets through these services can influence how frequently they are used. Anecdotally, food outlet access through online food delivery services increased in England between 2020 and 2022, in the context of the COVID-19 pandemic. However, the extent to which this access changed is poorly understood.

**Objective:**

We aimed to investigate monthly changes in online access to food prepared out of home in England in the context of the first 2 years of the COVID-19 pandemic compared with November 2019 and the extent to which any changes were associated with deprivation.

**Methods:**

In November 2019 and monthly between June 2020 and March 2022, we used automated data collection to construct a data set containing information about all food outlets in England registered to accept orders through the leading online food delivery service. Across postcode districts, we identified the number and percentage of food outlets registered to accept orders and the number that was accessible. We used generalized estimating equations (adjusted for population density, the number of food outlets in the physical food environment, and rural/urban classification) to investigate the change in outcomes compared with prepandemic levels (November 2019). We stratified analyses by deprivation quintile (Q).

**Results:**

Across England, the summed number of food outlets registered to accept orders online increased from 29,232 in November 2019 to 49,752 in March 2022. Across postcode districts, the median percentage of food outlets registered to accept orders online increased from 14.3 (IQR 3.8-26.0) in November 2019 to 24.0 (IQR 6.2-43.5) in March 2022. The median number of food outlets accessible online decreased from 63.5 (IQR 16.0-156.0) in November 2019 to 57.0 (IQR 11.0-163.0) in March 2022. However, we observed variation by deprivation. In March 2022, the median number of outlets accessible online was 175.0 (IQR 104.0-292.0) in the most deprived areas (Q5) compared with 27.0 (IQR 8.5-60.5) in the least deprived (Q1). In adjusted analyses, we estimated that the number of outlets accessible online in the most deprived areas was 10% higher in March 2022 compared with November 2019 (incidence rate ratios: 1.10, 95% CI 1.07-1.13). In the least deprived areas, we estimated a 19% decrease (incidence rate ratios: 0.81, 95% CI 0.79-0.83).

**Conclusions:**

The number of food outlets accessible online increased only in the most deprived areas in England. Future research might attempt to understand the extent to which changes in online food access were associated with changes in online food delivery service use and the possible implications on diet quality and health.

## Introduction

Purchasing and consuming food prepared out of home has become increasingly popular in many countries [1]. This food accounted for over 50% of total food expenditure in the United States in 2018 [2], and more than one-quarter of total food and beverage expenditure in the United Kingdom between 2015 and 2017 [3]. Food prepared out of home is often energy-dense [4,5], with the majority of items that are served by large chain restaurants exceeding recommended levels for sodium, fat, saturated fat, or sugars [6]. In turn, more frequent consumption of food prepared out of home has been positively associated with bodyweight [7]. Online food delivery services are now an established way of purchasing such food [8]. It is plausible, therefore, that the use of these services has implications for dietary practices, obesity, and health [9].

When using online food delivery services (such as UberEats), customers receive aggregated information about all food outlets that will deliver to them based on their location. Customers then select a food outlet and place and pay for their order on a single platform. Orders are forwarded to food outlets where meals are prepared before being delivered by couriers working for them or the online food delivery service [9]. As in the physical food environment [10], there is evidence that the number of food outlets accessible to individuals online (ie, the number that will deliver to them) can influence online food delivery service use. Among adults living in the United Kingdom, only those with the highest number of food outlets accessible online (between 182 and 879 outlets) self-reported any online food delivery service use in the previous week [11]. Even before the COVID-19 pandemic, the potential for this food-purchasing practice was not equally distributed across England, where the adjusted mean number of food outlets accessible online in 2019 was over 100 in the most deprived areas, compared with 70 in the least deprived areas [12]. This difference could contribute to known socioeconomic inequalities in diet-related health [13].

Government responses to the COVID-19 pandemic with respect to out of home food retail varied across countries worldwide. The Supplemental Nutrition Assistance Program was extended to include digital food retail in the United States [14], and the rules regarding the delivery of food prepared out of home and alcohol were relaxed in Australia [15]. In the United Kingdom, emergency regulations that allowed bars, cafés, pubs, and restaurants to offer a takeaway food service without specific permission were in place between March 2020 and March 2022 [16-18]. Further details are shown in Table 1.

**Table 1:** Details of selected emergency regulations introduced, action taken, and broader events in England in relation to the COVID-19 pandemic and food outlets selling food prepared out of home.

Time	Emergency regulations introduced, action taken, and broader events
Before March 2020	Food outlets in the physical food environment operated with a *primary use* based on their core business operations. The *primary use* of hot food takeaway outlets would be to serve food prepared out of home for off-premises consumption after previously receiving Local Authority^a^ planning permission to operate. Other establishments (bars, cafés, pubs, and restaurants) could offer a hot food takeaway service *in addition* to their primary use but would need Local Authority planning permission to do so. All food outlets with a customer-facing premises in the physical food environment could feasibly register to accept orders online regardless of their primary use without Local Authority planning permission^b^.
March 2020	Bars, cafés, pubs, and restaurants were forced to close for on-premises food consumption as part of *first* national lockdown^c^. Emergency regulations allowed bars, cafés, pubs, and restaurants to offer a hot food takeaway service in addition to their primary use introduced.
July 2020	Bars, cafés, pubs, and restaurants were allowed to reopen for on-premises food consumption but only with table service and with restricted capacity.
August 2020	The *Eat Out to Help Out* scheme that offered a 50% discount on meals, up to £10 (US $12) per person, every Monday, Tuesday, and Wednesday between August 3 and 31 was introduced for on-premises food consumption.
November 2020	Bars, cafés, pubs, and restaurants were forced to close for on-premises food consumption as part of *second* national lockdownc. Emergency regulations introduced in March 2020 were extended until March 2022.
December 2020	Bars, cafés, pubs, and restaurants were allowed to reopen for on-premises food consumption but only with table service and with restricted capacity.
January 2021	Bars, cafés, pubs, and restaurants were forced to close for on-premises food consumption as part of *third* national lockdownc.
April 2021	Bars, cafés, pubs, and restaurants were allowed to reopen for on-premises food consumption but only with table service and with restricted capacity.
July 2021	Restrictions necessitating table service only and capacity limits for on-premises food consumption inside bars, cafés, pubs, and restaurants ended.
March 2022	Emergency regulations introduced in March 2020 ended.
After March 2022	Bars, cafés, pubs, and restaurants *should* revert to their primary use and *should* stop offering a hot food takeaway service if adopted as part of emergency regulations. To continue offering an additional hot food takeaway service, Local Authority planning permission would be required. All food outlets with customer-facing premises in the physical food environment can feasibly register to accept orders online regardless of their primary use without Local Authority planning permission.

^a^Local Authorities are administrative bodies that operate at a subnational and subregional level.

^b^Registration to accept orders through online food delivery services remained viable regardless of any subsequent action or change.

^c^As part of lockdown orders in England, individuals were instructed to remain *at home*.

With regard to the link between the information outlined in Table 1 and online food delivery services, food sold through these services is typically prepared in the kitchens of food outlets that are customer facing in the physical food environment [9]. The location of these premises is socioeconomically distributed in England, with higher numbers and concentrations in more deprived areas [19]. As such, in more deprived areas, it is possible that a greater number of existing food outlets chose to operate within emergency regulations introduced during the COVID-19 pandemic and registered to accept orders online. In turn, this could influence absolute and relative levels of online food accessibility and aforementioned inequalities.

First, we aimed to investigate changes in levels of online access to food prepared out of home in England during the first 2 years of the COVID-19 pandemic. Second, we aimed to identify the extent to which any changes were associated with area-level deprivation.

## Methods

In this study, we built on our previous research that investigated cross-sectional associations between deprivation and online food outlet access in England [12]. Given that we have previously reported our methods in full, in this study we provide an overview.

### Study Setting, Period, and Analytic Scale

The setting for our study was England, and the study period was from November 2019 to March 2022, which coincided with the end of emergency regulations introduced during the COVID-19 pandemic (Table 1). Data were not collected between December 2019 and May 2020, meaning that first available data after November 2019 were from June 2020.

We completed analyses at the postcode district level because this reflects how food outlets registered to accept orders online delineated where they would deliver to in November 2019. Postcode districts are contained in the first half of full postcodes. For example, for the *postcode* CB2 0QQ, the *postcode district* is CB2. Postcode districts have an average size of 33 square miles [20] and a median population of 23,610 (IQR 13,320-34,560) [21].

We used postcode district boundary data from 2012, sourced from the United Kingdom Data Service [22], to map postcode districts in a Geographic Information System (GIS; ArcGIS version 10.7.1; ESRI Inc). We considered 2118 postcode districts eligible for inclusion in our analyses, reflecting those with boundaries entirely within or intersecting the border of England.

### Exposure Measure

We modeled our exposure measure (time) based on the frequency of data collection for our outcomes (monthly). We investigated changes in each of our outcomes over time compared with baseline (November 2019).

### Outcome Measures

#### Data Source and Collection

For each outcome, we collected data from an online food delivery service that was considered to be the UK market leader, Just Eat. In 2019, approximately 30,000 food outlets in the United Kingdom were registered to accept orders through this service, and there were approximately 170 million orders placed by customers [23,24]. In pilot analyses conducted in 2020, only a minority of food outlets registered to accept orders through Deliveroo (the next largest online food delivery service platform in the United Kingdom) were not also registered to accept orders through Just Eat [12]. Therefore, we collected data from Just Eat as a representation of online food delivery services. We refer to our data source as “the online food delivery service” hereafter.

In November 2019 and then monthly between June 2020 and March 2022, we used a web-browser extension to identify and collect the postcode of all food outlets in England that were registered to accept orders through the online food delivery service [25].

#### Number of Food Outlets Registered to Accept Orders Online

To accept orders through the online food delivery service, food outlets must register with them. In doing so, food outlets will have information about their business displayed on the online food delivery service platform irrespective of the location where they would deliver. We used Doogal (a free-to-use web service) to geocode the postcode of each food outlet identified during data collection [26] and excluded those that could not be geocoded (monthly range 0.08%-1.40%). We then mapped the locations of food outlets in our GIS based on supplied coordinates and counted the number located in each postcode district boundary.

#### Number of Food Outlets Accessible Online

In November 2019, June 2020, and July 2020, food outlets registered to accept orders online published the postcode districts that they would deliver to as their delivery areas. After July 2020, the online food delivery service no longer published this information. Therefore, between August 2020 and March 2022, after we searched the online food delivery service website by a given postcode district, we identified unique food outlets that appeared in our search results. We counted this number to determine those that would be accessible for a given population living in each postcode district (this is the number of food outlets that could be ordered from). Although we used 2 approaches, resultant data used in the outcome construction were the same.

#### Percentage of Food Outlets Registered to Accept Orders Online

We compared the number of food outlets registered to accept orders online with the number of outlets located in the physical food environment of the same postcode district. In doing so, we estimated the percentage of food outlets registered to accept orders online.

For the denominator (the number of outlets located in the physical food environment of a postcode district), we used the Ordnance Survey Points of Interest (OS POI) data set. These are commercial data containing information about retailers across multiple sectors, collated from more than 170 suppliers [27]. We extracted information for the following food outlet classifications: “Fast food and takeaway outlets” (food outlets selling food for off-premises consumption); “Fast food delivery services” (food outlets selling food for delivery, not explicitly online); “Fish and Chip shops” (food outlets selling a traditional British cuisine, typically for off-premises consumption); “Restaurants” (food outlets selling food for on-premises consumption); “Pubs, Bars, Inns” (establishments that primarily serve alcohol, that can also sell food for on-premises consumption); and “Cafe, Snack Bars & Tea Rooms” (food outlets selling food with no distinguishable consumption location). We selected these classifications based on a priori knowledge that they included food outlets typically registered to accept orders online and to reflect emergency regulations introduced during the COVID-19 pandemic (Table 1). We used coordinates supplied with OS POI data to map the locations of food outlets in our GIS. These coordinates have a stated accuracy of 1 meter [27].

We matched monthly data from the online food delivery service with OS POI data collected quarterly (Multimedia Appendix 1). We did not match individual food outlets listed in each data set, meaning that this outcome is the number of food outlets registered to accept orders online (based on data from the online food delivery service) as a percentage of the number of food outlets in the physical food environment (based on OS POI data set). Although we report a percentage, we acknowledge that in the strictest sense, we did not calculate it as such. We bounded this measure between 0% and 100% because the number of food outlets registered to accept orders online should not exceed the number of food outlets in the physical food environment. When the percentage exceeded 100%, it represented that a retailer not classified as a food outlet in OS POI data (according to our included classifications) was registered to accept orders online. We excluded postcode districts when this occurred (n=3).

#### Covariates

Food sold through online food delivery services is typically prepared in the kitchens of food outlets located in the physical food environment. Therefore, online food outlet access might be a function of physical food outlet access. We used OS POI data to account for the number of food outlets in the physical food environment when we did not use it as the denominator (ie, for the percentage of food outlets registered to accept orders online).

We used data from the 2019 Index of Multiple Deprivation to measure relative deprivation. This is a compound measure that includes metrics across the following domains: income deprivation, employment deprivation, crime, health deprivation and disability, education, skills and training deprivation, barriers to housing and services, and living environment deprivation [28]. Deprivation values are available for lower super output areas (LSOAs) in England, which are administrative boundaries with a mean residential population of 1500 people [21]. We calculated the mean Index of Multiple Deprivation value of LSOAs within, and intersecting, the boundary of each postcode district. For analyses, we split postcode districts into quintiles (Qs) of deprivation, with Q5 being the most deprived.

We used data from the 2011 rural/urban classification to categorize postcode districts as “rural,” when LSOAs within or intersecting their boundary were most frequently rural (populations <10,000 people within combined settlements, most of whom lived in rural-related areas), or “urban,” when intersecting LSOAs were most frequently urban (populations >10,000 people within combined settlements, most of whom lived in urban-related areas) [29]. We used data from the 2011 UK census [30] to identify the number of individuals who usually lived in a postcode district. Of the 2118 postcode districts, data for rural/urban classification and population density were available for 2097 (99%) and 2088 (95.4%) postcode districts, respectively, and did not change over the study period.

### Statistical Analysis

#### Overview

We used the longitudinal analysis (“xt”) suite of tools in Stata (version 16.1; StataCorp) to complete statistical analysis [31]. We report findings from the start (November 2019) to the end (March 2022) of the study period in the *Results* section. Findings for all time points (November 2019 and then monthly between June 2020 and March 2022) are available in Multimedia Appendix 1.

#### Descriptive Statistics

For each measure, we calculated the median (IQR) or the mean (SD) and the percentage change from baseline at each time point.

#### Inferential Statistics

We used generalized estimating equations (GEE) to investigate changes in each outcome over time (the exposure measure).

We completed a complete case analysis, whereby included postcode districts had complete data on all relevant measures. Data for count-based outcomes (the number of food outlets registered to accept orders online and the number of food outlets accessible online) were not normally distributed and were overdispersed. We used negative binomial GEE to account for this. Negative binomial GEE report incidence rate ratios (IRRs) and 95% CIs. In the context of this study, IRRs are the expected change in the outcome value at each time point compared with the baseline value (November 2019). For the percentage of food outlets registered to accept orders online, we rescaled the data to be between 0 and 1 and specified a binomial distribution [32]. Model coefficients for this outcome are the change at each time point compared with baseline values. We first completed unadjusted analyses and then analyses adjusted for covariates. For the number of food outlets registered to accept orders online and the number of food outlets accessible online, we included population density, rural/urban classification, and the number of food outlets in the physical food environment as covariates in our adjusted model. For the percentage of food outlets registered to accept orders online, we only included population density and rural/urban classification as covariates.

We report the findings from our adjusted models in the *Results* section, and our unadjusted models in Multimedia Appendix 1. From our adjusted models, we also estimated the mean count from IRRs and the mean percentage from coefficients and report these in the *Results* section. We report the respective IRRs and coefficients in Multimedia Appendix 1.

#### Associations With Deprivation

For each outcome, we included an interaction term between time and deprivation in our adjusted GEE and completed a post hoc test for significance (with statistical significance set at *P*<.01 to account for multiple testing). When interaction terms were significant, we completed analyses stratified by deprivation. In November 2019, there were inequalities in access to food outlets selling food prepared out of home online [12]. For each outcome, we calculated a slope index of inequality measure at baseline (November 2019) and at the end of the study period (March 2022) to investigate how inequalities changed over time. This measure of inequality is the difference in the respective outcome between the least and most deprived areas, estimated using linear regression [33,34].

### Ethical Considerations

Our study relied on publicly available data. Research ethics committee approval was not required.

## Results

### Overview

A descriptive summary of online food access in England is shown in Table 2 and Tables S1-S8 in Multimedia Appendix 1.

**Table 2:** Descriptive summary of online food accessibility at the postcode district level in England, stratified by deprivation quintile^a^.

				Deprivation quintile, median (IQR)											England, median (IQR)
				1 (least deprived)		2		3		4		5 (most deprived)			
**Number of food outlets registered to accept orders online^b^**															
	**Count**														
		November 2019	3.0 (1.0 to 8.0)		4.0 (1.0 to 12.0)		6.0 (1.0 to 18.0)		13.0 (3.0 to 25.0)		24.0 (12.0 to 39.0)		7.0 (1.0 to 21.0)		
		March 2022	5.0 (1.0 to 15.0)		8.0 (1.0 to 21.0)		10.0 (2.0 to 29.0)		21.0 (4.0 to 41.0)		35.0 (20.0 to 59.0)		13.0 (3.0 to 34.0)		
	**Change from baseline (%)^c^**														
		March 2022	80.0 (22.2 to 120.0)		69.0 (33.3 to 120.0)		66.7 (30.0 to 106.3)		62.8 (33.3 to 100.0)		57.9 (34.0 to 87.5)		65.4 (33.3 to 100.0)		
**Number of food outlets accessible online^d^**															
	**Count**														
		November 2019	37.0 (14.0 to 70.5)		38.0 (10.0 to 96.0)		62.0 (8.5 to 134.5)		86.0 (12.0 to 190.0)		164.0 (87.0 to 273.0)		63.5 (16.0 to 156.0)		
		March 2022	27.0 (8.5 to 60.5)		27.0 (6.0 to 103.0)		50.0 (6.5 to 133.5)		95.0 (13.0 to 217.0)		175.0 (104.0 to 292.0)		57.0 (11.0 to 163.0)		
	**Change from baseline (%)^c^**														
		March 2022	−12.7 (−48.6 to 20.0)		−7.5 (−48.8 to 28.6)		−1.1 (−35.0 to 33.2)		13.8 (−18.2 to 52.5)		13.1 (−7.9 to 33.8)		0.0 (−32.0 to 33.3)		
**Percentage of food outlets registered to accept orders online^e^**															
	**Percent (%)**														
		November 2019	7.9 (2.2 to 14.9)		8.7 (1.5 to 19.0)		12.5 (1.9 to 23.5)		20.4 (6.5 to 30.8)		27.8 (19.7 to 37.4)		14.3 (3.8 to 26.0)		
		March 2022	13.2 (4.7 to 25.9)		14.9 (4.1 to 33.3)		20.5 (5.3 to 36.6)		30.8 (11.3 to 46.6)		41.9 (30.7 to 52.4)		24.0 (7.7 to 41.0)		
	**Change from baseline (%)^c^**														
		March 2022	70.7 (16.7 to 116)		62.0 (25.0 to 106.2)		55.1 (24.0 to 98.9)		53.0 (24.8 to 88.9)		44.8 (22.4 to 75.8)		55.3 (23.0 to 96.1)		

^a^Data are reported as median (IQR); postcode districts are small geographical units used for mail routing in England.

^b^Food outlets in the physical food environment registered to accept orders through the UK market leading online food delivery service.

^c^Baseline=November 2019.

^d^Food outlets registered to accept orders through the UK marketing leading online food delivery service that would deliver to a given postcode district.

^e^Calculated as the number of food outlets registered to accept orders through the UK market leading online food delivery service compared with the number of the physical food environment.

### Number of Food Outlets Registered to Accept Orders Online

**Figure 1:**
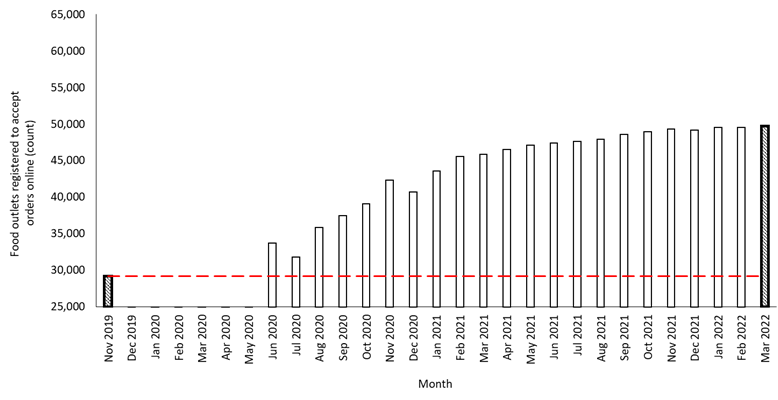
Summed number (count) of food outlets registered to accept orders online in England between November 2019 and March 2022. Shaded bars represent time points reported in the Results section: baseline (November 2019) and end (March 2022). No data were available from December 2019 to May 2020.

The summed number of food outlets registered to accept orders online in England increased from 29,232 in November 2019 to 49,752 in March 2022, equating to 70.2% growth (Figure 1).

**Figure 2:**
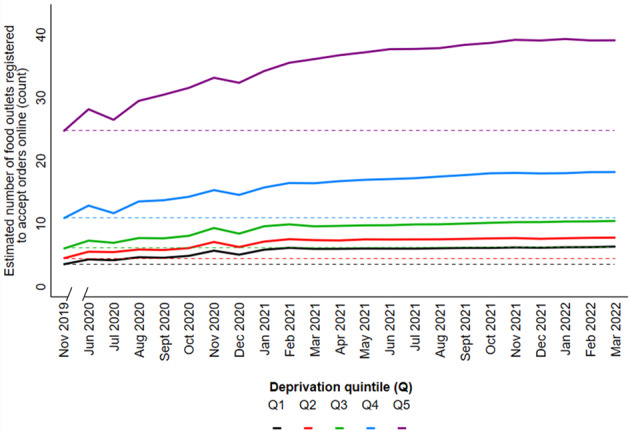
Estimated mean number (count) of food outlets registered to accept orders online in England, stratified by deprivation Q, where Q5 represents the most deprived postcode districts. Estimated number calculated from IRRs of a negative binomial generalized estimated equation adjusted for population density, rural/urban classification, and the number of food outlets in the physical food environment. In all, 2067 postcode districts were included. IRR: incidence rate ratio; Q: quintile.

The median number of food outlets registered to accept orders online per postcode district was 7.0 (IQR 1.0-21.0) in November 2019 and 13.0 (IQR 3.0-34.0) in March 2022. The median percent change from baseline (November 2019) per postcode district was 65.4 (IQR 33.3-100.0) in March 2022. The overall increase in the number of food outlets registered to accept orders was significant at each time point in our adjusted model (Table S10 in Multimedia Appendix 1 provides the IRRs), and there was significant effect modification by deprivation (*P*<.001). Estimated means calculated from IRRs of our adjusted negative binomial GEE are shown in Figure 2. At each level of deprivation, we observed that the estimated number of outlets registered to accept orders online had initially increased from baseline levels (November 2019); this decreased immediately after June 2020 and was then followed by a more consistent upward trend. The estimated number was consistently highest in the most deprived areas (Q5) and lowest in the least deprived (Q1). Absolute growth over time was also highest in the most deprived areas. For these areas, the estimated number was 39.1 outlets at the end of the study period, compared with 24.6 outlets at baseline, whereas for the least deprived areas, this was 6.6 and 3.5 outlets, respectively. The slope index of inequality between the least and most deprived areas was 5.0 (95% CI 4.5-4.5) outlets at baseline, and 7.8 (95% CI 7.0-8.6) outlets in March 2022.

### Number of Food Outlets Accessible Online

**Figure 3:**
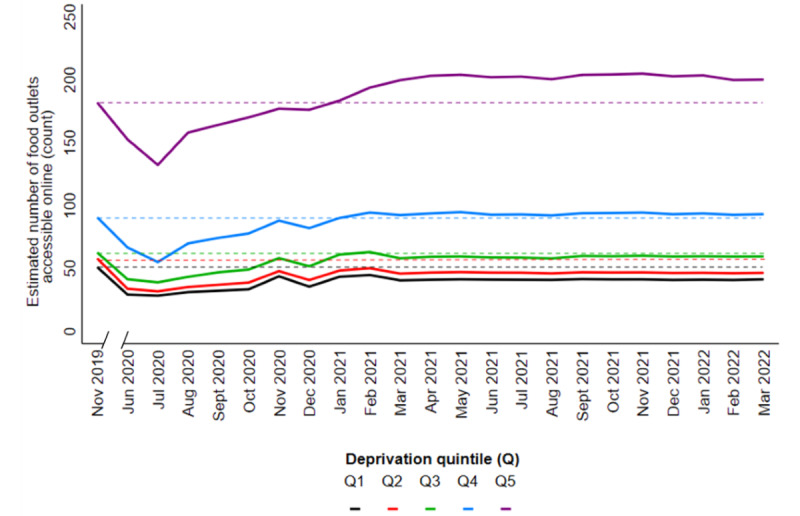
Estimated mean number (count) of food outlets accessible online in England, stratified by deprivation Q, where Q5 represents the most deprived postcode districts. Estimated number calculated from IRRs of a negative binomial generalized estimated equation adjusted for population density, rural/urban classification, and the number of food outlets in the physical food environment. In all, 2067 postcode districts were included. IRR: incidence rate ratio; Q: quintile.

The median number of food outlets accessible online per postcode district was 63.5 (IQR 16.0-156.0) in November 2019 and 57.0 (IQR 11.0-163.0) in March 2022. The median percent change from baseline per postcode district was 0.0 (IQR −32.0 to 33.3) in March 2022. The overall decrease in the number of food outlets accessible online was significant at each time point in our adjusted model (Table S12 in Multimedia Appendix 1 provides the IRRs), and there was a significant effect modification by deprivation (*P*<.001). Estimated means calculated from the IRRs of our adjusted negative binomial GEE are shown in Figure 3. At each level of deprivation, we observed that the estimated number of food outlets accessible online had decreased from baseline in June 2020. Although this was followed by an upward trajectory, the estimated number remained lower than baseline in less deprived areas (Q1-Q3) but surpassed baseline in those in the 2 upper Qs of deprivation (Q4 and Q5). For the most deprived areas (Q5), the estimated number increased from 181.9 outlets in November 2019 to 200.0 outlets in March 2022, and this contributed to an increasing gap in online food outlet access between the least and most deprived areas. The slope index of inequality between the least and most deprived areas was 32.0 (95% CI 28.1-35.9) outlets at baseline and 37.3 (95% CI 31.8-42.9) outlets in March 2022.

### Percentage of Food Outlets Registered to Accept Orders Online

**Figure 4:**
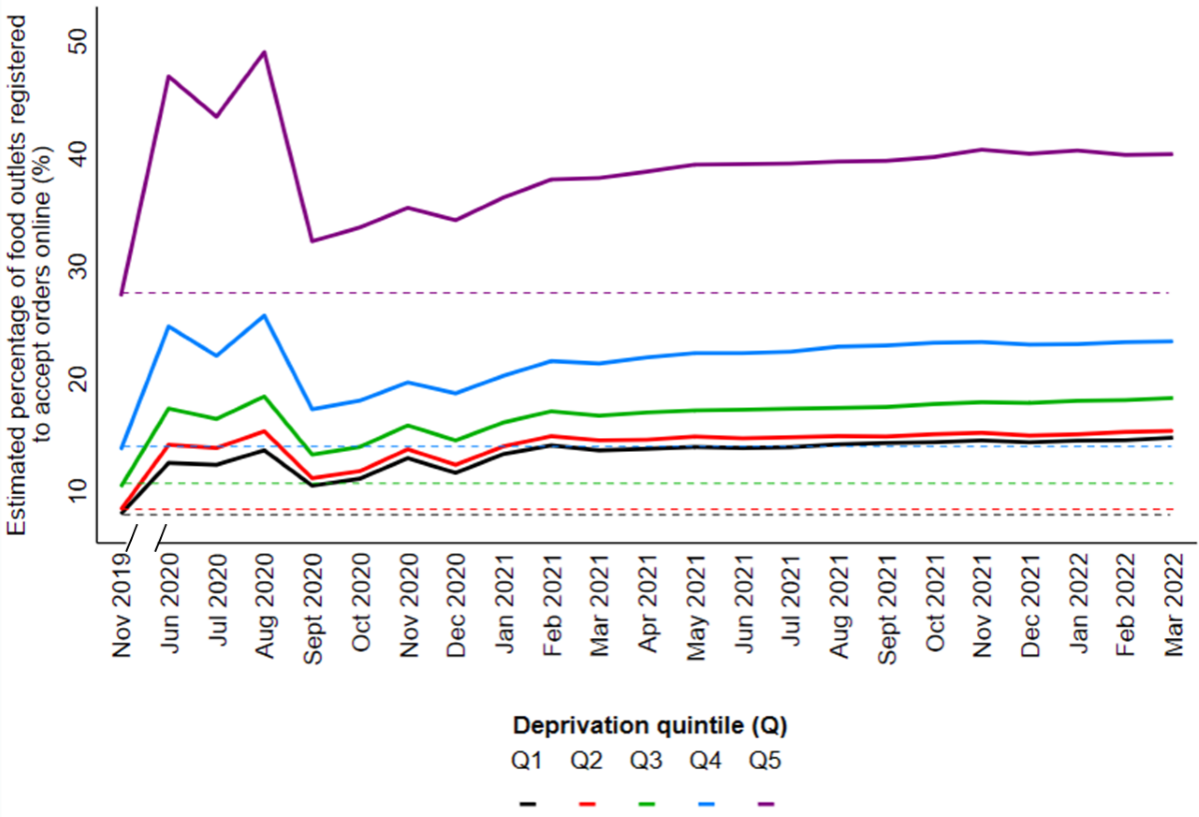
Estimated mean percentage (%) of food outlets registered to accept orders online in England, stratified by derivation Q, where Q5 represents the most deprived postcode districts. Estimated percentage calculated from coefficients of a generalized estimating equation adjusted for population density and rural/urban classification. In all, 2065 postcode districts were included. Q: quintile.

The median number of food outlets registered to accept orders online as a percentage of the number of food outlets in the physical food environment per postcode district was 14.3 (IQR 3.8-26.0) in November 2019 and 24.0 (IQR 6.2-43.5) in March 2022. The median percent change from baseline per postcode district was 55.3 (IQR 23.0-96.1) in March 2022. The overall increase in the percentage of food outlets registered to accept orders online was significant at each time point in our adjusted model (Table S14 in Multimedia Appendix 1 provides the coefficients), and there was a significant effect modification by deprivation (*P*<.001). Estimated means calculated from the coefficients of our adjusted GEE are shown in Figure 4. We observed an initial increase from baseline in June 2020 that was followed by a slight decline, a second increase that equaled or surpassed previous levels, and then another decline before a more stable increase. Although this trend was evident across all levels of deprivation and the estimated percentage was significantly increased at each level of deprivation by the end of the study period, the magnitude varied. Nevertheless, the estimated mean percentage was the highest in the most deprived areas (40.0% in March 2022 compared with 27.4% in November 2019), with these areas also having a higher growth over time in absolute terms, compared with other areas. The slope index of inequality between the least and most deprived areas was 4.5% (95% CI 4.1-4.9) at baseline and 5.9% (95% CI 5.4-6.5) in March 2022.

## Discussion

### Summary of Findings

To the best of our knowledge, our study was the first to examine changes in levels of online food access across an entire country during the COVID-19 pandemic. We identified an increase in the number of food outlets registered to accept orders online, reaching approximately 50,000 in England by March 2022. There was a parallel increase in the percentage of food outlets in the physical food environment registered to accept orders online. In contrast, the number of food outlets accessible online, that is, those that would deliver to a given population living in a postcode district, was on average lower in March 2022 than before the COVID-19 pandemic. The magnitude of change for all measures of online food access varied by level of deprivation. The most deprived postcode districts consistently had the highest number and percentage of food outlets registered to accept orders online. Importantly, contrary to the trend for England as a whole, only in the most deprived postcode districts was the number of food outlets accessible online higher in March 2022 than at baseline. We did not observe this for postcode districts at any other level of deprivation, with the number *decreasing* in less deprived areas. As a result, there is some evidence that existing socioeconomic inequalities in the opportunity to use online food delivery services to purchase food prepared out of home widened over time.

### Comparison With Prior Work

Our finding that an increased number of food outlets had registered to accept orders online in the first 2 years of the COVID-19 pandemic aligns with the reports presented in business and news media from major online food delivery services operating in England [35,36]. Moreover, the decrease in July 2020 accords with contemporaneous reports of food outlet closures and decreased order volume through online food delivery services [37]. For the most part, after July 2020, we observed an increase in the number of food outlets registered to accept orders online. As opportunities for on-premises food consumption were limited by national stay-at-home orders imposed in March 2020, followed by periods of time with restrictions on the capacity for on-premises dining, food outlet owners perhaps made a strategic business decision to register to accept orders online as a way to generate revenue.

For England as a whole, we identified an overall decline in the number of food outlets *accessible* online (ie, the number that will deliver to a given population living in a postcode district) between November 2019 and March 2022. This is in contrast to the increased number of food outlets *registered* to accept orders over the same period (irrespective of whom they would deliver to). The decline we observed was particularly pronounced in the early stages of the COVID-19 pandemic, which might have reflected a period of transition among those that had only recently registered to accept orders online. Food outlets unaccustomed to food delivery plausibly operated with a more limited delivery radius to ensure they could fulfill customer orders. Moreover, at this time, there was broader workforce capacity instability owing to self-isolation rules, unclear restrictions on maximum travel distances legally allowed, and concerns for online food delivery service courier safety [38]. All of which might have contributed to the implementation of limited delivery areas.

Although the initial decline in the number of food outlets accessible online was followed by an increase at all levels of deprivation, it was only among more deprived areas, and particularly the most deprived, that the number eventually surpassed prepandemic levels over the period of our study. The number of food outlets accessible online was already the highest in the most deprived areas in England before the pandemic [12]. However, we found evidence of an increasing divergence between the most and least deprived areas, suggesting that inequality in online food access widened during the period of our study. A perceived lack of demand in less deprived areas might have meant that registered food outlets chose not to include them in their delivery areas. Moreover, the delivery of food sold through online food delivery services is mostly completed by couriers on bicycles. As such, there is a natural limit in the distance that can be traveled while maintaining food quality. In rural, less deprived areas, this challenge might be insurmountable. Regardless, given that the number of food outlets accessible online is positively associated with online food delivery service use [11], it is possible that food-purchasing practices, diet, and health were negatively influenced during the COVID-19 pandemic, especially among populations living in more deprived areas. Although adults living in the United Kingdom self-reported a decrease in their diet quality during the COVID-19 pandemic [39], evidence on changes in the frequency of having food prepared out of home delivered is inconsistent [40]. Further research is required to understand the extent to which the changes in online food outlet access we observed were associated with changes in online food delivery service use during the same period and subsequent implications for diet and health. This future research could incorporate further measures of food access, for example, accounting for the affordability of food sold, which is recognized by online food delivery service customers as being an important consideration preceding use [41]. It is plausible that it is not financially viable for populations living in the most deprived areas to use online food delivery services despite the increase in the number of food outlets accessible, especially owing to the increase in the cost of living in the United Kingdom and elsewhere [42].

The number of food outlets registered to accept orders online as a percentage of the number of food outlets in the physical food environment had increased by the end of the study period. However, this increase was only apparent after a period of instability. Owners of food outlets with customer-facing premises in the physical food environment reported that although being registered to accept orders online was a way for customers to access their food when on-premises food consumption and travel was restricted, simultaneously managing orders placed in-person and online was difficult [38]. If food outlets that did not previously accept orders online only did so out of necessity when on-premises food consumption was restricted, it is plausible that they subsequently deregistered when restrictions ended. This scenario would partly explain the successive increases and decreases that coincided with the start and end of restrictions introduced during the COVID-19 pandemic (Figure 4).

Although the relative change over time with respect to the percentage of food outlets registered to accept orders online was consistent across all levels of deprivation, the absolute change was highest in the most deprived areas, which led to a widening of absolute inequality in online access to food prepared out of home. Food sold through online food delivery services is typically prepared in the kitchen facilities of food outlets located in the physical food environment [9]. Our finding is likely a reflection of the existing urban form in the most deprived areas in England, which is characterized by a high density and concentration of food outlets [43,44]. Although we cannot be certain, if more food outlets were operating within emergency regulations introduced during the COVID-19 pandemic, more outlets may have also registered to accept orders online. Our findings therefore provide evidence to suggest that the introduction of emergency regulations at least partly contributed to widening inequality in this exposure.

There remains considerable scope for the percentage of food outlets in the physical food environment that are registered to accept orders online to increase. This emphasizes the coexisting and overlapping nature of digital and physical food environments, which together provide multiple opportunities to purchase energy-dense, nutrient-poor food [45]. However, there are likely to be natural limits to growth because all food outlets do not necessarily need or want to register to accept orders online. In fact, the relative stability from June 2021 onward suggests that a plateau might have already occurred. An important limitation of this paper is that we do not have additional prepandemic data that would allow us to account for any existing trends in analyses. Nevertheless, our findings and associated data can contribute to future surveillance of longer-term trends.

### Possible Implications for Public Health and Policy

Urban planning has been used by over half of local authorities in England to promote the creation of healthier physical food environments, specifically by preventing new takeaway food outlets from opening [46]. To our knowledge, restrictions targeted specifically at online food delivery services are not in place. Although not yet fully clear, similar to the physical food environment, interventions to restrict access to food prepared out of home through these services might be increasingly necessary in the future [47,48]. At this point, public health interventions that do not necessarily attempt to restrict online food access but instead attempt to mitigate the potential public health burden posed by online food delivery service use possibly represent the most appropriate route to regulation. For example, changing the nutritional composition of food sold inside outlets would be expected to have a crossover effect and also change food sold online. Additionally, in England, in 2022, mandatory calorie labeling of non–prepacked food and soft drinks was introduced for retailers that operate with more than 250 employees, which meant that this information had to be displayed both inside food outlets and on the platforms of online food delivery services [49,50]. The introduction of this regulation recognizes the role of the digital food environment in the purchase of food prepared out of home. Research in the future could seek to further understand if and how regulations in the physical food environment can be extended to the digital food environment.

Furthermore, there has been evolution in the preparation location for the food sold through online food delivery services. This is best demonstrated by the development of facilities known as dark kitchens [51]. These facilities allow food businesses to register to accept orders through online food delivery services and prepare meals for delivery without the financial costs of having a customer-facing premises. To date, there is only limited evidence on the dark kitchen business model, which may not be generalizable beyond the data collection context of a single London borough [52]. Nevertheless, the development of these facilities plausibly influenced our findings related to the percentage of food outlets registered to accept orders online. Future research might seek to understand the public health implications of the dark kitchen business model, including how it uniquely contributes to the number of food outlets accessible online.

### Methodological Considerations and Limitations

Our data collection spanned 2 years and allowed us to closely monitor trends in important metrics of online food outlet access. In doing so, we present new baseline levels for future assessment. Nevertheless, our study is not without limitations. Our analysis was observational, and we cannot definitively conclude that the changes we observed were due to the COVID-19 pandemic. We also do not have sufficient prepandemic data that would allow underlying or existing trends to be accounted for in analyses. This is particularly the case for changes between November 2019 and June 2020 when we had no data, and we could not determine whether changes coincided with the COVID-19 pandemic.

We completed data collection at a single time point on a monthly basis. If food outlets were only registered to accept orders or accessible in the intervening period, they might not have been returned in our searches. However, the changes in the number of food outlets accessible online that we observed, especially the initial decrease in June 2020, is similar to reports of food outlet closures and decreased order volume at this time [37].

We did not track individual food outlets over the study period. Instead, we studied the total number of food outlets registered to accept orders at any given time point. This means that we are unable to comment on the number that were newly registered during the COVID-19 pandemic. It would be interesting for future work to investigate the extent to which food outlets that registered only during this period remained registered.

Our findings demonstrate *potential* online food outlet access. For this to be realized, any given individual must be an online food delivery service customer. As such, our findings do not necessarily translate into realized individual-level online food outlet access [53].

We used postcode districts as our unit of analysis and acknowledge the possibility that our findings are subject to the modifiable areal unit problem. Although the spatial unit that we adopted for analyses has the potential to introduce bias [54], our approach was data driven to allow consistency with our previous research [12]. Relatedly, using postcode districts as our unit of analysis also meant that we were limited to using boundary data from 2012. These data were not temporally aligned with further covariate data that were the most recently published or collected.

### Conclusions

We investigated changes in multiple measures of food access through online food delivery services during the first 2 years of the COVID-19 pandemic. We identified that the number of food outlets in England that were registered to accept orders online increased. In parallel, the number of food outlets registered to accept orders online as a percentage of the number of food outlets in the physical food environment increased. Although the number of food outlets that could be accessed decreased for the whole of England, trends differed by the level of deprivation. The number of outlets decreased compared with prepandemic levels in the least deprived areas and increased to surpass prepandemic levels *only* in the most deprived.

Overall, then, we identified considerable changes in measures of online food accessibility during a period when on-premises food consumption was often restricted. Our data from March 2022 represent a new baseline to which future changes in measures of online food accessibility can be investigated. Future research might attempt to understand the extent to which the changes we identified were associated with the changes in online food delivery service use and, in turn, the implications for diet quality and health.

## Appendix

Multimedia Appendix 1 Supplementary tables.

## Abbreviations

GEE: generalized estimating equations
GIS: Geographic Information System
IRR: incidence rate ratio
LSOA: lower super output area
OS POI: Ordnance Survey Points of Interest
Q: quintile

## Data Availability

The data that support the findings of this study were collected from sources available in the public domain: Just Eat Takeaway, the UK Census, the Indices of Multiple Deprivation, and UK boundary data. Data availability is subject to request and license restrictions. Further data were sourced from Digimap Edina, and we included data from PointX (c) DatabaseRight/Copyright (2022) and OS (c) Crown Copyright (2022). Data collected from Just Eat are accessible upon reasonable request; however, their use is for noncommercial purposes only.
