# Supplementary material for: Changes in Online Food Access During the COVID-19 Pandemic and Associations With Deprivation: Longitudinal Analysis
Source: JMIR Public Health Surveill. 2023 Apr 17;9:e41822. doi: 10.2196/41822 (PMC10131934; doi:10.2196/41822)
Supplement: Multimedia Appendix 1 [file publichealth_v9i1e41822_app1.docx]

| Supplementary Table 1: number (count) of food outlets registered to accept orders online in postcode districts in England, stratified by deprivation ^a b^. | | | | | | |
| --- | --- | --- | --- | --- | --- | --- |
|  | Deprivation quintile | | | | |  |
|  | 1 (least deprived) | 2 | 3 | 4 | 5 (most deprived) | England |
| **Month** |  |  |  |  |  |  |
| Nov 2019 | 3.0 (1.0-8.0) | 4.0 (1.0-12.0) | 6.0 (1.0-18.0) | 13.0 (3.0-25.0) | 24.0 (12.0-39.0) | 7.0 (1.0-21.0) |
| Jun 2020 | 4.0 (1.0-9.0) | 5.0 (1.0-15.0) | 7.0 (1.0-19.5) | 15.0 (3.0-28.0) | 25.0 (14.0-42.0) | 8.0 (2.0-24.0) |
| Jul 2020 | 3.0 (1.0-9.0) | 5.0 (0.0-15.0) | 6.0 (1.0-17.0) | 12.0 (2.0-26.0) | 24.0 (10.0-39.0) | 7.0 (1.0-22.0) |
| Aug 2020 | 4.0 (1.0-10.0) | 5.0 (1.0-16.0) | 7.0 (1.0-20.0) | 14.0 (3.0-29.0) | 27.0 (13.0-44.0) | 8.0 (2.0-25.0) |
| Sept 2020 | 4.0 (1.0-11.0) | 5.0 (1.0-16.0) | 7.0 (1.0-21.0) | 15.0 (4.0-30.0) | 28.0 (15.0-46.0) | 9.0 (2.0-26.0) |
| Oct 2020 | 4.0 (1.0-11.0) | 6.0 (1.0-16.0) | 7.0 (1.0-22.0) | 16.0 (4.0-32.0) | 29.0 (16.0-48.0) | 10.0 (2.0-27.0) |
| Nov 2020 | 5.0 (1.0-13.0) | 7.0 (1.0-19.0) | 9.0 (2.0-24.5) | 17.0 (4.0-34.0) | 30.0 (17.0-50.0) | 11.0 (3.0-29.0) |
| Dec 2020 | 4.0 (1.0-11.5) | 6.0 (1.0-17.0) | 8.0 (1.0-23.0) | 16.0 (4.0-33.0) | 30.0 (16.0-48.0) | 10.0 (2.0-28.0) |
| Jan 2021 | 5.0 (1.0-13.0) | 7.0 (1.0-20.0) | 10.0 (2.0-24.0) | 18.0 (4.0-35.0) | 31.0 (18.0-51.0) | 11.0 (3.0-30.0) |
| Feb 2021 | 5.0 (2.0-14.0) | 7.0 (1.0-20.0) | 10.0 (2.0-25.0) | 18.5 (5.0-38.0) | 33.0 (18.0-54.0) | 12.0 (3.0-31.0) |
| Mar 2021 | 5.0 (1.5-13.0) | 7.0 (1.0-21.0) | 9.0 (2.0-25.0) | 18.0 (4.0-38.0) | 33.0 (18.0-54.0) | 12.0 (3.0-32.0) |
| Apr 2021 | 5.0 (1.0-14.0) | 7.0 (1.0-20.0) | 9.5 (2.0-25.0) | 18.5 (4.0-38.0) | 33.0 (18.0-55.0) | 12.0 (3.0-32.0) |
| May 2021 | 5.0 (1.0-14.0) | 7.0 (1.0-20.0) | 9.5 (2.0-25.5) | 19.0 (5.0-39.0) | 34.0 (18.0-56.0) | 12.0 (3.0-32.0) |
| Jun 2021 | 5.0 (1.0-14.0) | 7.0 (1.0-19.0) | 10.0 (2.0-25.5) | 20.0 (5.0-39.0) | 35.0 (19.0-56.0) | 12.0 (3.0-32.0) |
| Jul 2021 | 5.0 (1.0-14.0) | 8.0 (1.0-19.0) | 10.0 (2.0-26.0) | 19.5 (5.0-39.0) | 35.0 (19.0-56.0) | 12.0 (3.0-33.0) |
| Aug 2021 | 5.0 (1.0-13.5) | 8.0 (1.0-20.0) | 10.0 (2.0-27.0) | 19.0 (4.0-41.0) | 34.0 (19.0-56.0) | 12.0 (3.0-33.0) |
| Sept 2021 | 5.0 (1.0-14.0) | 8.0 (1.0-20.0) | 10.0 (2.0-27.0) | 20.0 (4.0-41.0) | 35.0 (19.0-58.0) | 13.0 (3.0-33.0) |
| Oct 2021 | 5.0 (1.0-14.0) | 8.0 (1.0-21.0) | 10.0 (2.0-28.0) | 20.0 (5.0-41.0) | 35.0 (20.0-60.0) | 13.0 (3.0-33.0) |
| Nov 2021 | 5.0 (1.0-14.0) | 8.0 (1.0-21.0) | 10.0 (2.0-28.0) | 20.0 (5.0-41.0) | 36.0 (20.0-59.0) | 13.0 (3.0-34.0) |
| Dec 2021 | 5.0 (1.0-14.0) | 8.0 (1.0-20.0) | 10.0 (2.0-27.5) | 20.0 (5.0-41.0) | 35.0 (20.0-59.0) | 13.0 (3.0-34.0) |
| Jan 2022 | 5.0 (1.0-14.5) | 8.0 (1.0-21.0) | 10.0 (2.0-27.5) | 20.0 (4.0-41.0) | 36.0 (20.0-59.0) | 13.0 (3.0-34.0) |
| Feb 2022 | 5.0 (1.0-15.0) | 8.0 (1.0-21.0) | 10.0 (1.5-28.0) | 21.0 (5.0-41.0) | 36.0 (19.0-59.0) | 13.0 (3.0-34.0) |
| Mar 2022 | 5.0 (1.0-15.0) | 8.0 (1.0-21.0) | 10.0 (2.0-29.0) | 21.0 (4.0-41.0) | 35.0 (20.0-59.0) | 13.0 (3.0-34.0) |
| Note: ^a^ data are reported as Median (IQR).  ^b^ food outlets with a premises in the physical food environment registered to accept orders through the online food delivery service. | | | | | | |

| Supplementary Table 2: change (%) for the number of food outlets registered to accept orders online in postcode districts in England, stratified by deprivation ^a b^. | | | | | | | | |
| --- | --- | --- | --- | --- | --- | --- | --- | --- |
|  | | | Deprivation quintile | | |  | | |
|  | 1 (least deprived) | 2 | | 3 | 4 | | 5 (most deprived) | England |
| **Month** |  |  | |  |  | |  |  |
| Nov 2019 (baseline) | - | - | | - | - | | - | - |
| Jun 2020 | 14.3 (0.0-36.7) | 14.0 (0.0-35.4) | | 11.1 (0.0-30.8) | 12.0 (0.0-25.0) | | 11.1 (0.0-23.1) | 12.0 (0.0-29.5) |
| Jul 2020 | 13.3 (0.0-39.4) | 15.4 (0.0-40.0) | | 13.2 (0.0-33.3) | 8.3 (-5.6-26.3) | | 10.7 (0.0-23.8) | 11.8 (0.0-33.3) |
| Aug 2020 | 20.0 (0.0-50.0) | 23.7 (0.0-50.0) | | 19.3 (0.0-46.2) | 20.0 (0.0-40.9) | | 17.0 (3.7-33.3) | 20.0 (0.0-42.9) |
| Sept 2020 | 25.0 (0.0-54.9) | 25.0 (0.0-50.0) | | 22.2 (0.0-50.0) | 24.8 (3.8-41.7) | | 22.2 (8.0-36.7) | 23.1 (0.0-46.8) |
| Oct 2020 | 33.3 (0.0-66.7) | 28.6 (0.0-60.0) | | 26.7 (4.9-55.6) | 28.6 (8.3-50.0) | | 26.7 (12.0-41.7) | 28.1 (6.3-50.0) |
| Nov 2020 | 50.0 (9.1-100.0) | 50.0 (20.0-85.7) | | 43.7 (18.2-83.3) | 35.9 (16.7-66.7) | | 32.1 (16.9-50.0) | 41.5 (16.7-75.0) |
| Dec 2020 | 36.8 (0.0-75.0) | 37.7 (0.0-68.6) | | 34.9 (0.0-66.7) | 30.8 (9.1-56.3) | | 28.6 (14.3-47.5) | 33.3 (7.1-61.5) |
| Jan 2021 | 58.3 (14.3-100.0) | 50.0 (25.0-100.0) | | 50.0 (20.0-87.5) | 41.3 (17.6-74.2) | | 36.1 (21.4-56.3) | 45.1 (20.0-83.3) |
| Feb 2021 | 66.7 (25.0-100.0) | 59.7 (30.3-100.0) | | 50.0 (23.1-100.0) | 45.2 (26.2-77.8) | | 41.3 (26.7-63.6) | 50.0 (25.2-91.8) |
| Mar 2021 | 60.0 (20.0-100.0) | 56.8 (28.6-100.0) | | 50.0 (19.4-87.5) | 45.6 (21.4-80.0) | | 42.9 (27.3-65.5) | 50.0 (24.0-87.5) |
| Apr 2021 | 63.2 (20.0-100.0) | 55.8 (27.3-100.0) | | 50.0 (20.0-91.7) | 49.2 (24.0-83.3) | | 44.4 (28.6-67.5) | 50.0 (25.0-91.7) |
| May 2021 | 66.7 (25.0-100.0) | 60.0 (30.0-100.0) | | 50.0 (22.2-95.7) | 51.8 (24.0-87.5) | | 47.5 (30.2-72.4) | 52.0 (26.7-95.2) |
| Jun 2021 | 66.7 (20.0-100.0) | 57.1 (28.6-100.0) | | 50.0 (23.1-100.0) | 51.8 (23.1-89.5) | | 50.0 (33.3-72.4) | 54.5 (27.3-100.0) |
| Jul 2021 | 66.7 (20.0-100.0) | 59.0 (30.8-100.0) | | 50.0 (23.1-100.0) | 51.9 (25.0-89.5) | | 50.0 (32.7-74.5) | 54.5 (28.0-100.0) |
| Aug 2021 | 68.8 (20.0-100.0) | 60.0 (31.6-100.0) | | 52.8 (20.0-100.0) | 52.8 (27.3-96.0) | | 50.0 (33.3-80.8) | 54.5 (28.0-100.0) |
| Sept 2021 | 68.4 (22.2-109.1) | 62.5 (33.3-100.0) | | 56.3 (25.0-100.0) | 56.3 (31.3-97.1) | | 51.6 (33.3-83.3) | 57.9 (30.8-100.0) |
| Oct 2021 | 71.4 (21.4-110.0) | 63.2 (33.3-100.0) | | 56.9 (28.0-100.0) | 60.0 (32.1-96.8) | | 52.8 (34.5-83.3) | 60.0 (30.7-100.0) |
| Nov 2021 | 75.0 (25.0-114.3) | 63.8 (33.3-104.3) | | 56.5 (26.2-100.0) | 61.3 (33.3-100.0) | | 55.6 (36.6-87.5) | 61.1 (33.3-100.0) |
| Dec 2021 | 66.7 (20.0-116.7) | 63.8 (33.3-106.3) | | 58.6 (27.3-100.0) | 59.2 (33.3-100.0) | | 55.6 (35.7-84.4) | 60.0 (33.0-100.0) |
| Jan 2022 | 71.4 (20.0-120.0) | 66.1 (34.5-107.1) | | 60.0 (28.1-106.3) | 62.5 (32.1-100.0) | | 57.1 (35.0-87.5) | 62.5 (33.3-100.0) |
| Feb 2022 | 71.4 (22.2-116.7) | 66.7 (33.3-114.3) | | 61.6 (28.6-109.7) | 62.0 (31.8-100.0) | | 55.6 (36.2-85.7) | 62.5 (33.3-100.0) |
| Mar 2022 | 80.0 (22.2-120.0) | 69.0 (33.3-120.0) | | 66.7 (30.0-106.3) | 62.8 (33.3-100.0) | | 57.9 (34.0-87.5) | 65.4 (33.3-100.0) |
| Note: ^a^ data are reported as Median (IQR). ^b^ food outlets with a premises in the physical food environment registered to accept orders through the online food delivery service. | | | | | | | | |

| Supplementary Table 3: number (count) of food outlets accessible online in postcode districts in England, stratified by deprivation ^a b^. | | | | | | |
| --- | --- | --- | --- | --- | --- | --- |
|  | Deprivation quintile | | | | |  |
|  | 1 (least deprived) | 2 | 3 | 4 | 5 (most deprived) | England |
| **Month** |  |  |  |  |  |  |
| Nov 2019 | 37.0 (14.0-70.5) | 38.0 (10.0-96.0) | 62.0 (8.5-134.5) | 86.0 (12.0-190.0) | 164.0 (87.0-273.0) | 63.5 (16.0-156.0) |
| Jun 2020 | 19.0 (6.0-43.0) | 18.0 (5.0-68.0) | 36.5 (4.0-95.0) | 64.5 (9.0-152.0) | 134.0 (72.0-223.0) | 41.0 (9.0-120.0) |
| Jul 2020 | 17.0 (4.0-42.5) | 16.0 (3.0-63.0) | 30.0 (2.0-85.0) | 38.5 (1.0-122.0) | 115.0 (45.0-207.0) | 32.0 (4.0-104.0) |
| Aug 2020 | 20.0 (6.0-47.0) | 17.0 (3.0-68.0) | 33.0 (3.0-97.0) | 58.5 (3.0-156.0) | 137.0 (67.0-235.0) | 38.0 (6.0-122.0) |
| Sept 2020 | 20.0 (6.0-48.0) | 19.0 (5.0-74.0) | 40.0 (4.0-103.5) | 70.0 (11.0-166.0) | 145.0 (76.0-242.0) | 45.0 (9.0-131.0) |
| Oct 2020 | 21.0 (6.0-49.0) | 20.0 (5.0-80.0) | 41.0 (5.0-106.5) | 71.5 (11.0-171.0) | 149.0 (79.0-254.0) | 45.5 (9.0-135.0) |
| Nov 2020 | 24.5 (20.0-51.0) | 23.0 (11.0-86.0) | 42.5 (10.0-110.0) | 76.0 (20.0-177.0) | 157.0 (84.0-261.0) | 48.0 (20.0-141.0) |
| Dec 2020 | 22.0 (7.0-52.5) | 21.0 (5.0-83.0) | 42.5 (5.0-111.5) | 74.5 (12.0-179.0) | 156.0 (81.0-260.0) | 47.0 (10.0-141.0) |
| Jan 2021 | 24.0 (19.0-53.5) | 23.0 (10.0-84.0) | 43.5 (10.0-114.5) | 77.0 (17.0-185.0) | 164.0 (84.0-273.0) | 49.0 (18.0-146.0) |
| Feb 2021 | 26.0 (17.0-58.0) | 23.0 (11.0-92.0) | 48.0 (10.0-127.0) | 83.5 (19.0-193.0) | 171.0 (89.0-286.0) | 53.0 (18.0-155.0) |
| Mar 2021 | 27.0 (9.0-59.0) | 24.0 (6.0-95.0) | 49.0 (5.5-131.0) | 86.5 (14.0-204.0) | 175.0 (93.0-298.0) | 55.0 (11.0-161.0) |
| Apr 2021 | 26.5 (9.0-59.0) | 25.0 (6.0-97.0) | 50.5 (6.0-132.0) | 89.5 (14.0-204.0) | 178.0 (94.0-300.0) | 54.5 (11.0-164.0) |
| May 2021 | 27.0 (9.0-59.0) | 24.0 (6.0-97.0) | 49.5 (6.0-133.0) | 88.5 (15.0-205.0) | 178.0 (97.0-299.0) | 55.0 (11.0-165.0) |
| Jun 2021 | 26.0 (9.0-59.0) | 25.0 (7.0-99.0) | 49.5 (6.0-133.5) | 89.5 (14.0-212.0) | 181.0 (97.0-301.0) | 54.0 (11.0-167.0) |
| Jul 2021 | 27.0 (9.0-58.5) | 25.0 (7.0-99.0) | 48.0 (6.0-133.0) | 89.5 (13.0-207.0) | 180.0 (100.0-299.0) | 55.0 (11.0-166.0) |
| Aug 2021 | 27.0 (8.0-59.5) | 26.0 (6.0-100.0) | 48.5 (5.5-133.0) | 89.5 (13.0-208.0) | 176.0 (98.0-294.0) | 56.0 (11.0-164.0) |
| Sept 2021 | 27.0 (9.0-58.5) | 26.0 (7.0-98.0) | 48.5 (6.0-136.0) | 91.0 (13.0-214.0) | 180.0 (102.0-299.0) | 56.0 (12.0-167.0) |
| Oct 2021 | 26.0 (9.0-59.5) | 27.0 (6.0-100.0) | 49.0 (6.0-136.5) | 90.0 (14.0-213.0) | 181.0 (100.0-301.0) | 56.0 (12.0-166.0) |
| Nov 2021 | 26.0 (8.5-59.5) | 27.0 (7.0-102.0) | 49.0 (6.0-136.0) | 93.0 (14.0-211.0) | 185.0 (103.0-305.0) | 57.0 (12.0-168.0) |
| Dec 2021 | 26.0 (8.0-58.5) | 26.0 (7.0-98.0) | 50.0 (6.5-132.0) | 93.5 (13.0-211.0) | 180.0 (104.0-303.0) | 57.0 (11.0-164.0) |
| Jan 2022 | 26.5 (8.0-60.0) | 26.0 (6.0-100.0) | 50.0 (6.0-133.5) | 95.5 (15.0-210.0) | 178.0 (106.0-303.0) | 57.0 (11.0-165.0) |
| Feb 2022 | 26.5 (8.0-60.0) | 25.0 (6.0-100.0) | 50.0 (7.0-134.5) | 97.0 (13.0-214.0) | 174.0 (104.0-297.0) | 57.0 (12.0-164.0) |
| Mar 2022 | 27.0 (8.5-60.5) | 27.0 (6.0-103.0) | 50.0 (6.5-133.5) | 95.0 (13.0-217.0) | 175.0 (104.0-292.0) | 57.0 (11.0-163.0) |
| Note: ^a^ data are reported as Median (IQR). ^b^ food outlets registered to accept orders online that would deliver to a given postcode district. | | | | | | |

| Supplementary Table 4: change (%) for the number of food outlets accessible online in postcode districts in England, stratified by deprivation ^a b^. | | | | | | |
| --- | --- | --- | --- | --- | --- | --- |
|  | Deprivation quintile | | | | |  |
|  | 1 (least deprived) | 2 | 3 | 4 | 5 (most deprived) | England |
| **Month** |  |  |  |  |  |  |
| Nov 2019 (baseline) | - | - | - | - | - | - |
| Jun 2020 | -37.7 (-62.8--13.0) | -33.3 (-57.1--12.4) | -28.6 (-53.2--4.1) | -19.0 (-36.2-0.0) | -17.3 (-27.6--4.2) | -25.5 (-50.0--6.7) |
| Jul 2020 | -40.5 (-71.4--14.3) | -33.3 (-64.3--10.7) | -31.3 (-66.7--3.1) | -21.4 (-60.0-0.0) | -17.0 (-32.0--2.7) | -27.6 (-60.5--5.9) |
| Aug 2020 | -34.1 (-64.3--10.0) | -33.3 (-62.5--7.7) | -27.7 (-56.1-0.0) | -15.6 (-38.7-10.0) | -12.6 (-27.1-2.2) | -23.5 (-50.0-0.0) |
| Sept 2020 | -33.3 (-62.5--8.3) | -29.9 (-55.6--2.3) | -22.3 (-49.1-0.0) | -11.5 (-31.9-12.2) | -8.8 (-23.1-6.2) | -19.6 (-46.0-0.0) |
| Oct 2020 | -30.9 (-60.0--5.7) | -28.6 (-53.8-0.0) | -20.1 (-47.6-2.6) | -8.3 (-30.2-16.7) | -5.8 (-20.6-10.0) | -16.9 (-43.7-5.3) |
| Nov 2020 | -11.1 (-37.3-21.1) | -10.1 (-40.0-24.8) | -10.9 (-36.5-20.8) | 0.6 (-22.2-25.8) | -0.8 (-16.2-14.4) | -5.5 (-30.9-20.8) |
| Dec 2020 | -28.6 (-55.6-0.0) | -24.2 (-55.0-3.6) | -17.3 (-44.2-6.6) | 0.0 (-23.9-22.4) | -1.8 (-17.6-12.9) | -13.0 (-41.9-11.1) |
| Jan 2021 | -7.1 (-36.5-24.8) | -8.3 (-38.9-30.8) | -6.0 (-33.3-27.2) | 5.6 (-19.6-33.6) | 2.2 (-14.6-17.5) | -1.5 (-29.1-25.0) |
| Feb 2021 | -4.8 (-33.3-29.0) | 0.0 (-32.0-33.3) | 0.0 (-30.2-30.7) | 11.0 (-13.8-41.1) | 8.0 (-9.0-24.0) | 2.4 (-23.8-30.0) |
| Mar 2021 | -14.8 (-47.5-14.7) | -10.1 (-48.1-21.6) | -5.9 (-36.8-24.3) | 11.8 (-14.4-40.4) | 10.5 (-7.5-28.4) | 0.0 (-31.6-27.4) |
| Apr 2021 | -14.5 (-48.4-14.8) | -8.3 (-49.2-25.0) | -3.7 (-37.0-26.5) | 12.1 (-12.5-43.6) | 12.6 (-5.5-30.8) | 0.0 (-31.5-28.6) |
| May 2021 | -14.0 (-47.1-17.4) | -8.3 (-46.0-24.3) | -3.7 (-37.0-26.6) | 13.9 (-12.9-43.4) | 12.8 (-4.3-31.5) | 0.0 (-31.3-29.3) |
| Jun 2021 | -12.5 (-48.4-20.0) | -8.2 (-45.5-24.1) | -2.4 (-35.3-25.5) | 13.1 (-14.6-43.3) | 12.7 (-5.5-32.2) | 0.0 (-30.8-28.7) |
| Jul 2021 | -15.2 (-50.0-18.2) | -8.3 (-44.7-25.0) | -4.6 (-37.5-25.2) | 14.0 (-14.2-41.4) | 12.9 (-6.4-32.6) | 0.0 (-31.6-28.4) |
| Aug 2021 | -16.3 (-50.0-15.4) | -11.3 (-50.0-20.4) | -4.0 (-39.2-25.0) | 10.6 (-13.9-43.5) | 11.8 (-7.2-31.7) | 0.0 (-32.7-28.3) |
| Sept 2021 | -15.2 (-50.0-18.4) | -8.3 (-49.2-25.0) | -1.8 (-36.8-28.9) | 12.8 (-14.7-45.5) | 13.9 (-4.5-34.1) | 0.0 (-31.0-30.6) |
| Oct 2021 | -14.5 (-50.0-18.5) | -8.7 (-47.4-25.9) | -2.3 (-38.4-31.4) | 12.3 (-14.3-49.2) | 14.3 (-4.6-33.6) | 0.0 (-31.8-31.7) |
| Nov 2021 | -14.5 (-49.4-14.3) | -8.8 (-50.0-27.7) | 0.0 (-37.0-31.0) | 14.6 (-14.8-51.7) | 14.7 (-5.8-35.2) | 0.0 (-32.3-32.3) |
| Dec 2021 | -16.1 (-50.0-15.0) | -9.5 (-50.0-26.5) | -4.0 (-36.1-30.9) | 13.6 (-15.6-50.0) | 14.0 (-6.3-33.4) | 0.0 (-32.7-31.7) |
| Jan 2022 | -13.5 (-48.6-16.7) | -9.3 (-50.0-25.0) | -1.4 (-37.3-31.4) | 15.3 (-13.8-52.7) | 13.3 (-5.7-34.6) | 0.0 (-31.8-31.6) |
| Feb 2022 | -14.5 (-50.0-19.0) | -9.4 (-50.0-27.3) | -1.8 (-36.0-32.3) | 11.8 (-16.7-50.0) | 13.2 (-7.9-33.1) | 0.0 (-33.3-32.0) |
| Mar 2022 | -12.7 (-48.6-20.0) | -7.5 (-48.8-28.6) | -1.1 (-35.0-33.2) | 13.8 (-18.2-52.5) | 13.1 (-7.9-33.8) | 0.0 (-32.0-33.3) |
| Note: ^a^ data are reported as Median (IQR). ^b^ food outlets registered to accept orders online that would deliver to a given postcode district. | | | | | | |

| Supplementary Table 5: percentage of food outlets registered to accept orders online in postcode districts in England, stratified by deprivation ^a b^. | | | | | | | | |
| --- | --- | --- | --- | --- | --- | --- | --- | --- |
|  | | | Deprivation quintile | | |  | | |
|  | 1 (least deprived) | 2 | | 3 | 4 | | 5 (most deprived) | England |
| **Month** |  |  | |  |  | |  |  |
| Nov 2019 | 7.9 (2.2-14.9) | 8.7 (1.5-19.0) | | 12.5 (1.9-23.5) | 20.4 (6.5-30.8) | | 27.8 (19.7-37.4) | 14.3 (3.8-26.0) |
| Jun 2020 | 11.8 (3.3-21.5) | 13.6 (3.0-30.4) | | 19.0 (3.7-36.2) | 31.9 (8.8-50.0) | | 48.6 (34.5-63.5) | 22.2 (6.2-43.5) |
| Jul 2020 | 10.5 (2.8-21.1) | 13.0 (0.0-31.0) | | 17.5 (2.4-35.0) | 26.7 (5.3-47.6) | | 47.1 (29.8-61.3) | 20.0 (4.3-41.2) |
| Aug 2020 | 12.5 (3.9-23.3) | 13.6 (2.6-32.8) | | 19.2 (4.0-39.2) | 31.6 (7.7-53.7) | | 50.9 (36.0-66.7) | 22.7 (6.0-46.0) |
| Sept 2020 | 9.7 (2.9-18.8) | 11.1 (2.5-25.0) | | 15.5 (3.4-28.6) | 23.7 (7.7-37.8) | | 33.3 (23.6-43.7) | 17.4 (5.2-32.7) |
| Oct 2020 | 10.5 (3.5-19.6) | 11.8 (2.9-26.0) | | 15.9 (3.8-29.8) | 24.0 (7.7-38.5) | | 34.2 (24.7-45.5) | 18.2 (5.6-33.8) |
| Nov 2020 | 12.5 (4.2-22.9) | 14.3 (3.2-29.7) | | 18.1 (4.9-33.8) | 25.8 (9.1-41.5) | | 36.0 (26.6-46.7) | 20.8 (6.8-36.6) |
| Dec 2020 | 10.8 (3.7-20.5) | 12.1 (2.6-27.0) | | 16.4 (3.8-31.4) | 24.2 (7.7-39.3) | | 35.1 (25.7-45.5) | 18.6 (5.7-34.8) |
| Jan 2021 | 12.5 (4.8-23.5) | 14.9 (3.0-29.8) | | 18.3 (4.7-33.8) | 26.3 (8.5-42.2) | | 37.4 (27.2-48.1) | 21.1 (6.9-37.5) |
| Feb 2021 | 13.5 (5.5-25.0) | 14.9 (3.4-32.3) | | 19.0 (5.0-36.0) | 27.9 (9.4-43.6) | | 39.2 (28.6-50.0) | 22.0 (7.4-39.1) |
| Mar 2021 | 12.5 (5.2-23.8) | 14.8 (3.4-31.3) | | 18.8 (4.3-34.3) | 27.0 (9.2-44.6) | | 39.5 (28.4-50.0) | 21.6 (7.1-38.6) |
| Apr 2021 | 12.5 (4.9-23.9) | 14.8 (3.0-31.3) | | 18.8 (4.6-34.0) | 27.7 (8.8-45.5) | | 40.2 (28.7-50.0) | 21.6 (7.1-39.2) |
| May 2021 | 12.4 (5.0-23.7) | 14.3 (3.4-32.6) | | 19.3 (4.9-34.4) | 28.4 (9.3-46.2) | | 40.9 (29.3-50.9) | 22.1 (7.2-40.0) |
| Jun 2021 | 12.5 (4.8-23.7) | 14.3 (3.9-32.4) | | 19.5 (4.9-34.6) | 27.9 (9.6-45.6) | | 40.7 (29.3-51.8) | 22.2 (7.1-40.0) |
| Jul 2021 | 12.5 (4.5-24.8) | 14.3 (4.0-32.6) | | 20.0 (4.9-34.6) | 28.1 (9.6-46.2) | | 40.7 (29.1-51.3) | 22.2 (7.1-40.0) |
| Aug 2021 | 12.5 (4.5-23.8) | 14.0 (3.9-33.3) | | 19.8 (4.9-35.3) | 29.8 (9.5-45.7) | | 41.0 (29.3-52.8) | 22.6 (7.1-40.3) |
| Sept 2021 | 13.0 (4.8-24.8) | 14.3 (3.9-32.9) | | 20.0 (4.9-35.0) | 29.4 (9.7-46.1) | | 40.9 (29.5-51.5) | 22.9 (7.2-40.3) |
| Oct 2021 | 13.0 (4.9-24.5) | 14.8 (3.9-33.3) | | 20.2 (5.3-35.8) | 29.5 (9.7-46.1) | | 41.2 (29.8-52.0) | 23.0 (7.5-40.8) |
| Nov 2021 | 13.1 (5.1-24.9) | 14.9 (4.0-33.3) | | 20.0 (5.3-35.9) | 30.1 (9.7-46.2) | | 41.5 (30.8-53.1) | 23.2 (7.4-40.9) |
| Dec 2021 | 12.5 (4.8-25.2) | 14.8 (4.0-33.3) | | 20.1 (5.3-35.6) | 30.0 (9.4-46.2) | | 40.9 (30.2-53.1) | 23.1 (7.2-40.8) |
| Jan 2022 | 12.7 (4.8-25.5) | 14.8 (4.0-33.0) | | 20.4 (5.0-36.2) | 30.1 (10.6-46.4) | | 41.5 (30.8-53.2) | 23.4 (7.4-40.9) |
| Feb 2022 | 12.8 (4.8-25.0) | 14.8 (4.2-33.3) | | 20.7 (5.3-36.6) | 31.0 (11.0-46.2) | | 41.5 (30.2-53.1) | 23.3 (7.7-40.8) |
| Mar 2022 | 13.2 (4.7-25.9) | 14.9 (4.1-33.3) | | 20.5 (5.3-36.6) | 30.8 (11.3-46.6) | | 41.9 (30.7-52.4) | 24.0 (7.7-41.0) |
| Note: ^a^  data are reported as Median (IQR). ^b^ percentage calculated as the number of food outlets registered to accept orders online compared to number of food outlets in the physical food environment. | | | | | | | | |

| Supplementary Table 6: change in percentage (%) for the percentage of food outlets registered to accept orders online in postcode districts in England, stratified by deprivation ^a b^. | | | | | | |
| --- | --- | --- | --- | --- | --- | --- |
|  | Deprivation quintile | | | | |  |
|  | 1 (least deprived) | 2 | 3 | 4 | 5 (most deprived) | England |
| **Month** |  |  |  |  |  |  |
| Nov 2019 (baseline) | - | - | - | - | - | - |
| Jun 2020 | 44.7 (17.4-81.0) | 54.1 (28.3-79.2) | 53.5 (30.0-82.9) | 62.9 (33.3-92.4) | 75.3 (49.2-99.5) | 58.1 (31.1-88.8) |
| Jul 2020 | 45.0 (14.8-83.9) | 56.6 (26.3-84.1) | 55.3 (22.2-84.6) | 55.5 (20.9-88.5) | 69.6 (40.6-99.7) | 57.2 (24.0-90.5) |
| Aug 2020 | 56.5 (20.8-95.7) | 63.6 (31.7-101.6) | 66.1 (37.5-101.3) | 74.1 (34.0-111.1) | 85.1 (52.8-115.3) | 69.2 (35.0-107.2) |
| Sept 2020 | 22.1 (0.0-54.6) | 21.3 (2.3-52.3) | 19.9 (2.3-44.0) | 22.1 (3.6-40.0) | 19.4 (3.4-36.6) | 20.5 (2.2-44.0) |
| Oct 2020 | 28.7 (0.0-62.5) | 26.8 (5.7-59.1) | 25.6 (5.2-55.2) | 25.4 (6.4-46.3) | 23.1 (7.7-39.4) | 25.2 (5.2-51.0) |
| Nov 2020 | 50.0 (9.0-100.0) | 46.9 (16.7-79.3) | 40.0 (13.9-76.5) | 34.3 (13.6-59.2) | 29.5 (14.1-50.7) | 38.3 (13.8-69.7) |
| Dec 2020 | 33.8 (1.8-71.4) | 33.5 (6.2-64.5) | 31.9 (5.9-60.9) | 27.3 (7.5-52.3) | 24.7 (8.0-43.8) | 29.2 (6.2-57.5) |
| Jan 2021 | 52.9 (10.2-105.7) | 50.9 (20.9-89.7) | 44.6 (17.1-82.9) | 38.2 (13.1-66.7) | 33.3 (15.2-53.8) | 41.3 (15.5-77.3) |
| Feb 2021 | 63.1 (23.3-113.5) | 57.1 (26.5-99.0) | 46.8 (20.0-88.6) | 41.2 (20.8-71.6) | 37.9 (20.6-59.5) | 46.3 (21.9-85.8) |
| Mar 2021 | 55.6 (19.3-102.3) | 51.6 (21.7-94.0) | 45.1 (14.5-79.8) | 43.5 (17.8-70.9) | 37.6 (20.2-61.3) | 44.6 (18.7-79.2) |
| Apr 2021 | 58.7 (20.3-100.0) | 50.3 (23.0-94.9) | 45.6 (17.9-83.8) | 44.4 (19.0-76.3) | 39.5 (20.6-63.0) | 45.6 (20.0-82.8) |
| May 2021 | 61.4 (19.3-103.9) | 53.0 (23.1-100.0) | 45.2 (19.1-86.6) | 47.5 (19.9-81.4) | 41.6 (22.8-66.1) | 48.4 (20.8-85.7) |
| Jun 2021 | 62.5 (15.4-105.6) | 51.8 (22.5-100.0) | 46.8 (18.6-88.7) | 45.0 (20.0-79.7) | 43.8 (23.8-65.0) | 48.6 (20.4-86.7) |
| Jul 2021 | 62.7 (12.8-103.0) | 53.0 (22.8-100.0) | 46.8 (18.6-89.7) | 46.5 (19.7-81.5) | 43.1 (23.8-66.7) | 49.2 (20.4-87.4) |
| Aug 2021 | 63.9 (13.3-105.8) | 54.0 (23.0-100.0) | 46.7 (14.3-89.6) | 47.0 (22.2-84.7) | 43.9 (24.2-67.3) | 50.0 (20.8-89.6) |
| Sept 2021 | 64.8 (16.7-109.8) | 54.2 (25.0-105.3) | 49.5 (17.2-89.3) | 46.7 (22.6-83.0) | 43.4 (24.3-70.2) | 50.5 (21.8-90.0) |
| Oct 2021 | 66.7 (15.3-109.1) | 55.9 (25.6-105.3) | 50.0 (21.0-90.9) | 50.0 (23.4-86.2) | 44.0 (25.4-73.0) | 51.9 (22.9-91.4) |
| Nov 2021 | 67.6 (18.4-112.3) | 56.9 (28.0-106.5) | 50.0 (19.1-90.9) | 51.8 (26.2-86.7) | 46.2 (24.4-76.3) | 53.0 (23.3-93.8) |
| Dec 2021 | 62.3 (14.5-111.9) | 58.2 (23.5-102.2) | 51.4 (22.6-92.0) | 49.1 (26.2-86.8) | 44.8 (23.4-74.5) | 51.9 (22.5-92.2) |
| Jan 2022 | 63.0 (15.3-112.3) | 58.9 (26.1-102.2) | 53.1 (23.0-100.0) | 53.6 (22.0-87.9) | 46.3 (24.0-76.3) | 53.8 (22.7-93.8) |
| Feb 2022 | 66.6 (16.7-113.5) | 58.6 (25.0-104.5) | 54.3 (21.9-100.0) | 54.2 (22.7-84.2) | 44.8 (25.0-74.8) | 54.2 (23.1-93.9) |
| Mar 2022 | 70.7 (16.7-116.0) | 62.0 (25.0-106.2) | 55.1 (24.0-98.9) | 53.0 (24.8-88.9) | 44.8 (22.4-75.8) | 55.3 (23.0-96.1) |
| Note: ^a^ data are reported as Median (IQR). Percentage calculated as the number of food outlets registered to accept orders online compared to number of food outlets in the physical food environment. | | | | | | |

| Supplementary Table 7: number (count) of food outlets in the physical food environment in postcode districts in England, stratified by deprivation ^a b^. | | | | | | |
| --- | --- | --- | --- | --- | --- | --- |
|  | Deprivation quintile | | | | |  |
|  | 1 (least deprived) | 2 | 3 | 4 | 5 (most deprived) | England |
| **Month** |  |  |  |  |  |  |
| Nov 2019 | 52.3 (42.0) | 59.5 (42.7) | 69.3 (65.4) | 83.3 (77.1) | 100.5 (82.1) | 71.9 (65.3) |
| Jun 2020 | 40.6 (29.9) | 44.6 (29.3) | 50.1 (42.9) | 56.7 (47.9) | 63.0 (48.5) | 50.5 (40.8) |
| Jul 2020 | 40.6 (29.9) | 44.6 (29.3) | 50.1 (42.9) | 56.7 (47.9) | 63.0 (48.5) | 50.5 (40.8) |
| Aug 2020 | 40.6 (29.9) | 44.6 (29.3) | 50.1 (42.9) | 56.7 (47.9) | 63.0 (48.5) | 50.5 (40.8) |
| Sept 2020 | 52.8 (42.5) | 60.2 (43.2) | 70.4 (66.4) | 84.9 (77.7) | 102.6 (82.1) | 73.1 (65.9) |
| Oct 2020 | 52.8 (42.5) | 60.2 (43.2) | 70.4 (66.4) | 84.9 (77.7) | 102.6 (82.1) | 73.1 (65.9) |
| Nov 2020 | 52.8 (42.5) | 60.2 (43.2) | 70.4 (66.4) | 84.9 (77.7) | 102.6 (82.1) | 73.1 (65.9) |
| Dec 2020 | 53.0 (42.9) | 60.6 (43.5) | 70.9 (66.9) | 85.2 (78.1) | 103.1 (82.2) | 73.5 (66.2) |
| Jan 2021 | 53.0 (42.9) | 60.6 (43.5) | 70.9 (66.9) | 85.2 (78.1) | 103.1 (82.2) | 73.5 (66.2) |
| Feb 2021 | 53.0 (42.9) | 60.6 (43.5) | 70.9 (66.9) | 85.2 (78.1) | 103.1 (82.2) | 73.5 (66.2) |
| Mar 2021 | 53.5 (43.2) | 61.2 (43.7) | 71.4 (67.0) | 86.1 (78.5) | 104.3 (83.0) | 74.2 (66.7) |
| Apr 2021 | 53.5 (43.2) | 61.2 (43.7) | 71.4 (67.0) | 86.1 (78.5) | 104.3 (83.0) | 74.2 (66.7) |
| May 2021 | 53.5 (43.2) | 61.2 (43.7) | 71.4 (67.0) | 86.1 (78.5) | 104.3 (83.0) | 74.2 (66.7) |
| Jun 2021 | 53.8 (43.5) | 61.4 (43.9) | 71.6 (67.3) | 86.6 (78.3) | 105.3 (83.2) | 74.6 (66.9) |
| Jul 2021 | 53.8 (43.5) | 61.4 (43.9) | 71.6 (67.3) | 86.6 (78.3) | 105.3 (83.2) | 74.6 (66.9) |
| Aug 2021 | 53.8 (43.5) | 61.4 (43.9) | 71.6 (67.3) | 86.6 (78.3) | 105.3 (83.2) | 74.6 (66.9) |
| Sept 2021 | 53.8 (43.7) | 61.8 (44.4) | 72.1 (68.1) | 87.7 (80.0) | 106.5 (84.1) | 75.3 (67.8) |
| Oct 2021 | 53.8 (43.7) | 61.8 (44.4) | 72.1 (68.1) | 87.7 (80.0) | 106.5 (84.1) | 75.3 (67.8) |
| Nov 2021 | 53.8 (43.7) | 61.8 (44.4) | 72.1 (68.1) | 87.7 (80.0) | 106.5 (84.1) | 75.3 (67.8) |
| Dec 2021 | 54.1 (43.9) | 62.1 (44.6) | 72.4 (68.3) | 88.1 (80.3) | 106.9 (84.3) | 75.6 (68.0) |
| Jan 2022 | 54.1 (43.9) | 62.1 (44.6) | 72.4 (68.3) | 88.1 (80.3) | 106.9 (84.3) | 75.6 (68.0) |
| Feb 2022 | 54.1 (43.9) | 62.1 (44.6) | 72.4 (68.3) | 88.1 (80.3) | 106.9 (84.3) | 75.6 (68.0) |
| Mar 2022 | 54.1 (43.9) | 62.1 (44.6) | 72.4 (68.3) | 88.1 (80.3) | 106.9 (84.3) | 75.6 (68.0) |
| Note: ^a^ data are reported as Mean (SD). ^b^ food outlets physically located in a postcode district, based on Ordnance Survey’s Points of Interest dataset. | | | | | | |

| Supplementary Table 8: change in number (%) for the number of food outlets in the physical food environment in postcode districts in England, stratified by deprivation ^a b^. | | | | | | |
| --- | --- | --- | --- | --- | --- | --- |
|  | Deprivation quintile | | | | |  |
|  | 1 (least deprived) | 2 | 3 | 4 | 5 (most deprived) | England |
| **Month** |  |  |  |  |  |  |
| Nov 2019 (baseline) | - | - | - | - | - | - |
| Jun 2020 | -18.4 (10.5) | -20.9 (11.0) | -22.8 (11.4) | -26.8 (13.1) | -33.4 (11.6) | -24.2 (12.6) |
| Jul 2020 | -18.4 (10.5) | -20.9 (11.0) | -22.8 (11.4) | -26.8 (13.1) | -33.4 (11.6) | -24.2 (12.6) |
| Aug 2020 | -18.4 (10.5) | -20.9 (11.0) | -22.8 (11.4) | -26.8 (13.1) | -33.4 (11.6) | -24.2 (12.6) |
| Sept 2020 | 1.0 (9.0) | 1.1 (8.0) | 1.7 (9.6) | 2.5 (10.1) | 4.0 (11.7) | 2.0 (9.7) |
| Oct 2020 | 1.0 (9.0) | 1.1 (8.0) | 1.7 (9.6) | 2.5 (10.1) | 4.0 (11.7) | 2.0 (9.7) |
| Nov 2020 | 1.0 (9.0) | 1.1 (8.0) | 1.7 (9.6) | 2.5 (10.1) | 4.0 (11.7) | 2.0 (9.7) |
| Dec 2020 | 1.4 (9.5) | 1.7 (8.2) | 2.6 (9.6) | 2.9 (11.3) | 4.7 (11.7) | 2.6 (10.1) |
| Jan 2021 | 1.4 (9.5) | 1.7 (8.2) | 2.6 (9.6) | 2.9 (11.3) | 4.7 (11.7) | 2.6 (10.1) |
| Feb 2021 | 1.4 (9.5) | 1.7 (8.2) | 2.6 (9.6) | 2.9 (11.3) | 4.7 (11.7) | 2.6 (10.1) |
| Mar 2021 | 2.2 (10.3) | 2.8 (9.0) | 3.3 (9.9) | 4.4 (12.7) | 6.0 (12.4) | 3.7 (10.9) |
| Apr 2021 | 2.2 (10.3) | 2.8 (9.0) | 3.3 (9.9) | 4.4 (12.7) | 6.0 (12.4) | 3.7 (10.9) |
| May 2021 | 2.2 (10.3) | 2.8 (9.0) | 3.3 (9.9) | 4.4 (12.7) | 6.0 (12.4) | 3.7 (10.9) |
| Jun 2021 | 2.6 (10.6) | 3.1 (9.1) | 3.6 (10.0) | 5.1 (12.9) | 7.3 (13.2) | 4.3 (11.3) |
| Jul 2021 | 2.6 (10.6) | 3.1 (9.1) | 3.6 (10.0) | 5.1 (12.9) | 7.3 (13.2) | 4.3 (11.3) |
| Aug 2021 | 2.6 (10.6) | 3.1 (9.1) | 3.6 (10.0) | 5.1 (12.9) | 7.3 (13.2) | 4.3 (11.3) |
| Sept 2021 | 2.5 (11.2) | 3.6 (10.2) | 4.1 (10.6) | 6.1 (13.3) | 8.5 (13.9) | 4.9 (12.0) |
| Oct 2021 | 2.5 (11.2) | 3.6 (10.2) | 4.1 (10.6) | 6.1 (13.3) | 8.5 (13.9) | 4.9 (12.0) |
| Nov 2021 | 2.5 (11.2) | 3.6 (10.2) | 4.1 (10.6) | 6.1 (13.3) | 8.5 (13.9) | 4.9 (12.0) |
| Dec 2021 | 3.1 (11.6) | 4.1 (10.3) | 4.5 (10.7) | 6.6 (13.5) | 9.0 (14.1) | 5.3 (12.2) |
| Jan 2022 | 3.1 (11.6) | 4.1 (10.3) | 4.5 (10.7) | 6.6 (13.5) | 9.0 (14.1) | 5.3 (12.2) |
| Feb 2022 | 3.1 (11.6) | 4.1 (10.3) | 4.5 (10.7) | 6.6 (13.5) | 9.0 (14.1) | 5.3 (12.2) |
| Mar 2022 | 3.1 (11.6) | 4.1 (10.3) | 4.5 (10.7) | 6.6 (13.5) | 9.0 (14.1) | 5.3 (12.2) |
| Note: ^a^ data are reported as Mean (SD). ^b^ food outlets physically located in a postcode district, based on Ordnance Survey Points of Interest dataset. | | | | | | |

| Supplementary Table 9: Incidence Rate Ratios (IRR) and 95% confidence intervals (CI) for the number (count) of food outlets registered to accept orders online in postcode districts in England, stratified by deprivation, estimated using an unadjusted negative binomial generalised estimating equation. | | | | | | | | | | | | | | | | | | |
| --- | --- | --- | --- | --- | --- | --- | --- | --- | --- | --- | --- | --- | --- | --- | --- | --- | --- | --- |
|  | Deprivation quintile | | | | | | | | | | | | | | |  | | |
|  | 1 (least deprived) | | | 2 |  |  | 3 |  |  | 4 |  |  | 5 (most deprived) | | | England | | |
|  | IRR | 95% CI | | IRR | 95% CI | | IRR | 95% CI | | IRR | 95% CI | | IRR | 95% CI | | IRR | 95% CI | |
| **Month** |  |  | |  |  | |  |  | |  |  | |  |  | |  |  | |
| Nov 2019 | ref | ref | ref | ref | ref | ref | ref | ref | ref | ref | ref | ref | ref | ref | ref | ref | ref | ref |
| Jun 2020 | 1.18 | 1.16 | 1.20 | 1.18 | 1.16 | 1.20 | 1.17 | 1.15 | 1.19 | 1.16 | 1.13 | 1.18 | 1.13 | 1.11 | 1.16 | 1.15 | 1.14 | 1.16 |
| Jul 2020 | 1.18 | 1.16 | 1.20 | 1.17 | 1.15 | 1.19 | 1.10 | 1.08 | 1.12 | 1.03 | 1.01 | 1.06 | 1.06 | 1.04 | 1.08 | 1.09 | 1.08 | 1.10 |
| Aug 2020 | 1.29 | 1.27 | 1.32 | 1.27 | 1.25 | 1.30 | 1.23 | 1.21 | 1.25 | 1.22 | 1.19 | 1.25 | 1.19 | 1.17 | 1.22 | 1.23 | 1.22 | 1.24 |
| Sept 2020 | 1.32 | 1.30 | 1.35 | 1.31 | 1.29 | 1.34 | 1.29 | 1.27 | 1.32 | 1.29 | 1.26 | 1.32 | 1.25 | 1.22 | 1.27 | 1.28 | 1.27 | 1.29 |
| Oct 2020 | 1.38 | 1.36 | 1.41 | 1.38 | 1.35 | 1.40 | 1.36 | 1.33 | 1.38 | 1.35 | 1.32 | 1.38 | 1.29 | 1.27 | 1.32 | 1.34 | 1.32 | 1.35 |
| Nov 2020 | 1.57 | 1.54 | 1.60 | 1.55 | 1.52 | 1.58 | 1.50 | 1.47 | 1.52 | 1.44 | 1.41 | 1.48 | 1.36 | 1.33 | 1.39 | 1.45 | 1.43 | 1.46 |
| Dec 2020 | 1.45 | 1.42 | 1.47 | 1.45 | 1.42 | 1.48 | 1.43 | 1.40 | 1.46 | 1.40 | 1.37 | 1.43 | 1.33 | 1.31 | 1.36 | 1.39 | 1.38 | 1.40 |
| Jan 2021 | 1.61 | 1.58 | 1.64 | 1.59 | 1.56 | 1.62 | 1.53 | 1.50 | 1.55 | 1.49 | 1.45 | 1.52 | 1.41 | 1.38 | 1.44 | 1.49 | 1.48 | 1.50 |
| Feb 2021 | 1.69 | 1.66 | 1.72 | 1.66 | 1.63 | 1.69 | 1.62 | 1.59 | 1.65 | 1.55 | 1.52 | 1.59 | 1.46 | 1.43 | 1.50 | 1.56 | 1.54 | 1.57 |
| Mar 2021 | 1.68 | 1.65 | 1.71 | 1.67 | 1.64 | 1.70 | 1.61 | 1.58 | 1.64 | 1.56 | 1.53 | 1.60 | 1.49 | 1.46 | 1.52 | 1.57 | 1.56 | 1.58 |
| Apr 2021 | 1.69 | 1.66 | 1.72 | 1.67 | 1.64 | 1.70 | 1.63 | 1.60 | 1.66 | 1.59 | 1.56 | 1.63 | 1.52 | 1.48 | 1.55 | 1.59 | 1.58 | 1.60 |
| May 2021 | 1.71 | 1.68 | 1.74 | 1.69 | 1.66 | 1.73 | 1.65 | 1.62 | 1.68 | 1.62 | 1.58 | 1.66 | 1.53 | 1.50 | 1.57 | 1.61 | 1.60 | 1.62 |
| Jun 2021 | 1.72 | 1.69 | 1.75 | 1.70 | 1.67 | 1.73 | 1.65 | 1.62 | 1.68 | 1.63 | 1.60 | 1.67 | 1.55 | 1.52 | 1.59 | 1.62 | 1.61 | 1.64 |
| Jul 2021 | 1.73 | 1.70 | 1.76 | 1.71 | 1.67 | 1.74 | 1.66 | 1.63 | 1.69 | 1.64 | 1.60 | 1.68 | 1.56 | 1.52 | 1.59 | 1.63 | 1.62 | 1.64 |
| Aug 2021 | 1.75 | 1.72 | 1.78 | 1.72 | 1.69 | 1.75 | 1.67 | 1.63 | 1.70 | 1.66 | 1.62 | 1.70 | 1.56 | 1.53 | 1.60 | 1.64 | 1.63 | 1.65 |
| Sept 2021 | 1.77 | 1.74 | 1.80 | 1.74 | 1.71 | 1.77 | 1.68 | 1.65 | 1.71 | 1.68 | 1.65 | 1.72 | 1.58 | 1.55 | 1.62 | 1.66 | 1.65 | 1.68 |
| Oct 2021 | 1.78 | 1.75 | 1.81 | 1.75 | 1.72 | 1.78 | 1.70 | 1.66 | 1.73 | 1.70 | 1.66 | 1.74 | 1.59 | 1.56 | 1.63 | 1.67 | 1.66 | 1.69 |
| Nov 2021 | 1.80 | 1.77 | 1.83 | 1.76 | 1.73 | 1.80 | 1.71 | 1.68 | 1.74 | 1.71 | 1.67 | 1.75 | 1.61 | 1.58 | 1.65 | 1.69 | 1.67 | 1.70 |
| Dec 2021 | 1.79 | 1.76 | 1.82 | 1.75 | 1.72 | 1.78 | 1.71 | 1.68 | 1.74 | 1.70 | 1.66 | 1.74 | 1.61 | 1.57 | 1.64 | 1.68 | 1.67 | 1.70 |
| Jan 2022 | 1.81 | 1.78 | 1.84 | 1.76 | 1.73 | 1.80 | 1.73 | 1.69 | 1.76 | 1.71 | 1.67 | 1.75 | 1.62 | 1.58 | 1.65 | 1.70 | 1.68 | 1.71 |
| Feb 2022 | 1.82 | 1.79 | 1.85 | 1.76 | 1.73 | 1.80 | 1.73 | 1.70 | 1.76 | 1.71 | 1.68 | 1.76 | 1.61 | 1.57 | 1.64 | 1.69 | 1.68 | 1.71 |
| Mar 2022 | 1.84 | 1.81 | 1.88 | 1.78 | 1.75 | 1.81 | 1.75 | 1.71 | 1.78 | 1.72 | 1.68 | 1.76 | 1.61 | 1.57 | 1.64 | 1.70 | 1.69 | 1.72 |

| Supplementary Table 10: Incidence Rate Ratios (IRR) and 95% confidence intervals (CI) for the number (count) of food outlets registered to accept orders online in postcode districts in England, stratified by deprivation, estimated using an adjusted negative binomial generalised estimating equation ^a^. | | | | | | | | | | | | | | | | | | |
| --- | --- | --- | --- | --- | --- | --- | --- | --- | --- | --- | --- | --- | --- | --- | --- | --- | --- | --- |
|  | Deprivation quintile | | | | | | | | | | | | | | |  | | |
|  | 1 (least deprived) | | | 2 |  |  | 3 |  |  | 4 |  |  | 5 (most deprived) | | | England | | |
|  | IRR | 95% CI | | IRR | 95% CI | | IRR | 95% CI | | IRR | 95% CI | | IRR | 95% CI | | IRR | 95% CI | |
| **Month** |  |  | |  |  | |  |  | |  |  | |  |  | |  |  | |
| Nov 2019 | ref | ref | ref | ref | ref | ref | ref | ref | ref | ref | ref | ref | ref | ref | ref | ref | ref | ref |
| Jun 2020 | 1.22 | 1.17 | 1.28 | 1.24 | 1.19 | 1.28 | 1.21 | 1.16 | 1.26 | 1.19 | 1.14 | 1.23 | 1.14 | 1.11 | 1.17 | 1.18 | 1.16 | 1.19 |
| Jul 2020 | 1.19 | 1.14 | 1.24 | 1.22 | 1.18 | 1.27 | 1.16 | 1.11 | 1.21 | 1.08 | 1.04 | 1.12 | 1.07 | 1.05 | 1.10 | 1.11 | 1.10 | 1.13 |
| Aug 2020 | 1.32 | 1.26 | 1.38 | 1.31 | 1.27 | 1.36 | 1.28 | 1.23 | 1.34 | 1.25 | 1.20 | 1.30 | 1.20 | 1.16 | 1.23 | 1.24 | 1.22 | 1.26 |
| Sept 2020 | 1.30 | 1.24 | 1.35 | 1.30 | 1.25 | 1.34 | 1.27 | 1.22 | 1.33 | 1.27 | 1.22 | 1.31 | 1.24 | 1.21 | 1.27 | 1.26 | 1.25 | 1.28 |
| Oct 2020 | 1.38 | 1.32 | 1.44 | 1.36 | 1.32 | 1.41 | 1.34 | 1.29 | 1.40 | 1.32 | 1.27 | 1.37 | 1.28 | 1.25 | 1.31 | 1.32 | 1.31 | 1.34 |
| Nov 2020 | 1.61 | 1.55 | 1.68 | 1.58 | 1.53 | 1.64 | 1.55 | 1.48 | 1.61 | 1.41 | 1.36 | 1.47 | 1.35 | 1.31 | 1.38 | 1.47 | 1.45 | 1.50 |
| Dec 2020 | 1.43 | 1.38 | 1.50 | 1.40 | 1.35 | 1.45 | 1.40 | 1.34 | 1.46 | 1.34 | 1.30 | 1.39 | 1.31 | 1.28 | 1.35 | 1.36 | 1.34 | 1.38 |
| Jan 2021 | 1.66 | 1.59 | 1.73 | 1.60 | 1.54 | 1.65 | 1.59 | 1.53 | 1.66 | 1.45 | 1.40 | 1.51 | 1.39 | 1.35 | 1.42 | 1.51 | 1.49 | 1.53 |
| Feb 2021 | 1.74 | 1.67 | 1.82 | 1.68 | 1.62 | 1.74 | 1.64 | 1.58 | 1.71 | 1.52 | 1.47 | 1.57 | 1.44 | 1.41 | 1.48 | 1.57 | 1.55 | 1.60 |
| Mar 2021 | 1.70 | 1.63 | 1.77 | 1.65 | 1.59 | 1.71 | 1.59 | 1.53 | 1.66 | 1.52 | 1.46 | 1.57 | 1.47 | 1.43 | 1.50 | 1.56 | 1.54 | 1.58 |
| Apr 2021 | 1.70 | 1.63 | 1.77 | 1.64 | 1.58 | 1.69 | 1.60 | 1.54 | 1.67 | 1.55 | 1.49 | 1.60 | 1.49 | 1.45 | 1.53 | 1.57 | 1.55 | 1.60 |
| May 2021 | 1.71 | 1.64 | 1.79 | 1.67 | 1.61 | 1.73 | 1.62 | 1.55 | 1.68 | 1.57 | 1.51 | 1.62 | 1.51 | 1.47 | 1.55 | 1.59 | 1.57 | 1.62 |
| Jun 2021 | 1.71 | 1.64 | 1.78 | 1.67 | 1.61 | 1.73 | 1.62 | 1.56 | 1.69 | 1.58 | 1.52 | 1.63 | 1.53 | 1.49 | 1.57 | 1.60 | 1.58 | 1.63 |
| Jul 2021 | 1.71 | 1.64 | 1.78 | 1.67 | 1.61 | 1.73 | 1.64 | 1.58 | 1.71 | 1.59 | 1.53 | 1.65 | 1.53 | 1.49 | 1.57 | 1.61 | 1.59 | 1.63 |
| Aug 2021 | 1.72 | 1.65 | 1.80 | 1.67 | 1.62 | 1.73 | 1.64 | 1.58 | 1.71 | 1.61 | 1.56 | 1.67 | 1.54 | 1.50 | 1.57 | 1.62 | 1.60 | 1.64 |
| Sept 2021 | 1.74 | 1.67 | 1.81 | 1.69 | 1.63 | 1.75 | 1.66 | 1.60 | 1.73 | 1.64 | 1.58 | 1.70 | 1.56 | 1.52 | 1.60 | 1.64 | 1.62 | 1.66 |
| Oct 2021 | 1.74 | 1.67 | 1.81 | 1.71 | 1.65 | 1.77 | 1.68 | 1.62 | 1.75 | 1.66 | 1.60 | 1.72 | 1.57 | 1.53 | 1.61 | 1.65 | 1.63 | 1.68 |
| Nov 2021 | 1.76 | 1.69 | 1.84 | 1.72 | 1.66 | 1.78 | 1.70 | 1.63 | 1.77 | 1.67 | 1.61 | 1.73 | 1.59 | 1.55 | 1.63 | 1.67 | 1.65 | 1.69 |
| Dec 2021 | 1.75 | 1.68 | 1.82 | 1.69 | 1.63 | 1.76 | 1.70 | 1.63 | 1.77 | 1.66 | 1.60 | 1.72 | 1.59 | 1.55 | 1.62 | 1.66 | 1.64 | 1.69 |
| Jan 2022 | 1.77 | 1.69 | 1.84 | 1.71 | 1.65 | 1.77 | 1.72 | 1.65 | 1.79 | 1.66 | 1.60 | 1.72 | 1.59 | 1.56 | 1.63 | 1.67 | 1.65 | 1.70 |
| Feb 2022 | 1.77 | 1.70 | 1.85 | 1.73 | 1.67 | 1.79 | 1.72 | 1.65 | 1.79 | 1.68 | 1.62 | 1.74 | 1.59 | 1.55 | 1.63 | 1.68 | 1.66 | 1.70 |
| Mar 2022 | 1.80 | 1.73 | 1.88 | 1.73 | 1.67 | 1.80 | 1.73 | 1.66 | 1.80 | 1.68 | 1.62 | 1.74 | 1.59 | 1.55 | 1.63 | 1.69 | 1.66 | 1.71 |
| Note: ^a^ adjusted for population density, rural urban classification and the number of food outlets in the physical food environment. | | | | | | | | | | | | | | | | | | |

| Supplementary Table 11: Incidence Rate Ratios (IRR) and 95% confidence intervals (CI) for the number (count) of food outlets accessible online in postcode districts in England, stratified by deprivation, estimated using an unadjusted negative binomial generalised estimating equation. | | | | | | | | | | | | | | | | | | |
| --- | --- | --- | --- | --- | --- | --- | --- | --- | --- | --- | --- | --- | --- | --- | --- | --- | --- | --- |
|  | Deprivation quintile | | | | | | | | | | | | | | |  | | |
|  | 1 (least deprived) | | | 2 |  |  | 3 |  |  | 4 |  |  | 5 (most deprived) | | | England | | |
|  | IRR | 95% CI | | IRR | 95% CI | | IRR | 95% CI | | IRR | 95% CI | | IRR | 95% CI | | IRR | 95% CI | |
| **Month** |  |  | |  |  | |  |  | |  |  | |  |  | |  |  | |
| Nov 2019 | ref | ref | ref | ref | ref | ref | ref | ref | ref | ref | ref | ref | ref | ref | ref | ref | ref | ref |
| Jun 2020 | 0.71 | 0.70 | 0.73 | 0.82 | 0.80 | 0.84 | 0.77 | 0.76 | 0.79 | 0.84 | 0.82 | 0.86 | 0.82 | 0.80 | 0.85 | 0.80 | 0.80 | 0.81 |
| Jul 2020 | 0.68 | 0.67 | 0.69 | 0.75 | 0.74 | 0.77 | 0.72 | 0.70 | 0.73 | 0.68 | 0.66 | 0.70 | 0.71 | 0.69 | 0.73 | 0.71 | 0.70 | 0.71 |
| Aug 2020 | 0.78 | 0.76 | 0.79 | 0.87 | 0.85 | 0.89 | 0.82 | 0.80 | 0.83 | 0.89 | 0.86 | 0.91 | 0.86 | 0.83 | 0.88 | 0.85 | 0.84 | 0.86 |
| Sept 2020 | 0.81 | 0.79 | 0.82 | 0.96 | 0.94 | 0.98 | 0.89 | 0.87 | 0.91 | 0.97 | 0.94 | 1.00 | 0.91 | 0.89 | 0.94 | 0.92 | 0.91 | 0.92 |
| Oct 2020 | 0.84 | 0.82 | 0.85 | 1.01 | 0.99 | 1.03 | 0.94 | 0.92 | 0.96 | 1.02 | 0.99 | 1.05 | 0.95 | 0.92 | 0.97 | 0.96 | 0.95 | 0.97 |
| Nov 2020 | 0.95 | 0.93 | 0.96 | 1.09 | 1.07 | 1.11 | 1.00 | 0.98 | 1.02 | 1.08 | 1.05 | 1.11 | 0.98 | 0.95 | 1.01 | 1.02 | 1.01 | 1.03 |
| Dec 2020 | 0.89 | 0.88 | 0.91 | 1.07 | 1.05 | 1.10 | 1.00 | 0.98 | 1.02 | 1.08 | 1.05 | 1.11 | 0.98 | 0.95 | 1.01 | 1.01 | 1.00 | 1.02 |
| Jan 2021 | 0.96 | 0.94 | 0.98 | 1.12 | 1.09 | 1.14 | 1.04 | 1.01 | 1.06 | 1.12 | 1.09 | 1.15 | 1.02 | 0.99 | 1.05 | 1.05 | 1.04 | 1.06 |
| Feb 2021 | 1.01 | 0.99 | 1.03 | 1.18 | 1.15 | 1.20 | 1.09 | 1.07 | 1.11 | 1.17 | 1.14 | 1.20 | 1.08 | 1.04 | 1.11 | 1.11 | 1.10 | 1.12 |
| Mar 2021 | 1.00 | 0.98 | 1.02 | 1.19 | 1.16 | 1.21 | 1.11 | 1.09 | 1.13 | 1.20 | 1.17 | 1.23 | 1.11 | 1.08 | 1.14 | 1.13 | 1.12 | 1.14 |
| Apr 2021 | 1.02 | 1.00 | 1.04 | 1.21 | 1.18 | 1.23 | 1.13 | 1.11 | 1.15 | 1.22 | 1.19 | 1.26 | 1.13 | 1.10 | 1.17 | 1.15 | 1.14 | 1.16 |
| May 2021 | 1.02 | 1.01 | 1.04 | 1.22 | 1.20 | 1.25 | 1.14 | 1.12 | 1.17 | 1.24 | 1.21 | 1.27 | 1.13 | 1.10 | 1.17 | 1.16 | 1.15 | 1.17 |
| Jun 2021 | 1.01 | 0.99 | 1.03 | 1.19 | 1.16 | 1.21 | 1.12 | 1.10 | 1.14 | 1.21 | 1.18 | 1.25 | 1.13 | 1.09 | 1.16 | 1.14 | 1.13 | 1.15 |
| Jul 2021 | 1.02 | 1.00 | 1.04 | 1.19 | 1.17 | 1.22 | 1.12 | 1.10 | 1.15 | 1.22 | 1.19 | 1.25 | 1.13 | 1.10 | 1.16 | 1.14 | 1.13 | 1.15 |
| Aug 2021 | 1.01 | 1.00 | 1.03 | 1.19 | 1.16 | 1.21 | 1.11 | 1.09 | 1.13 | 1.21 | 1.18 | 1.25 | 1.12 | 1.09 | 1.15 | 1.14 | 1.12 | 1.15 |
| Sept 2021 | 1.03 | 1.01 | 1.05 | 1.21 | 1.19 | 1.24 | 1.14 | 1.12 | 1.17 | 1.24 | 1.21 | 1.28 | 1.14 | 1.10 | 1.17 | 1.16 | 1.15 | 1.17 |
| Oct 2021 | 1.03 | 1.01 | 1.05 | 1.20 | 1.18 | 1.23 | 1.14 | 1.11 | 1.16 | 1.24 | 1.20 | 1.27 | 1.14 | 1.11 | 1.17 | 1.16 | 1.14 | 1.17 |
| Nov 2021 | 1.03 | 1.01 | 1.05 | 1.21 | 1.18 | 1.24 | 1.14 | 1.12 | 1.16 | 1.24 | 1.21 | 1.27 | 1.14 | 1.11 | 1.18 | 1.16 | 1.15 | 1.17 |
| Dec 2021 | 1.02 | 1.00 | 1.04 | 1.20 | 1.17 | 1.22 | 1.13 | 1.10 | 1.15 | 1.22 | 1.19 | 1.26 | 1.13 | 1.10 | 1.16 | 1.15 | 1.13 | 1.16 |
| Jan 2022 | 1.02 | 1.00 | 1.04 | 1.20 | 1.18 | 1.23 | 1.13 | 1.11 | 1.16 | 1.23 | 1.20 | 1.26 | 1.13 | 1.10 | 1.17 | 1.15 | 1.14 | 1.16 |
| Feb 2022 | 1.01 | 0.99 | 1.03 | 1.18 | 1.15 | 1.21 | 1.11 | 1.09 | 1.13 | 1.21 | 1.18 | 1.24 | 1.11 | 1.08 | 1.14 | 1.13 | 1.12 | 1.14 |
| Mar 2022 | 1.02 | 1.00 | 1.04 | 1.18 | 1.16 | 1.21 | 1.11 | 1.09 | 1.14 | 1.21 | 1.18 | 1.24 | 1.11 | 1.08 | 1.15 | 1.13 | 1.12 | 1.15 |

| Supplementary Table 12: Incidence Rate Ratios (IRR) and 95% confidence intervals (CI) for the number (count) of food outlets accessible online in postcode districts in England, stratified by deprivation, estimated using an adjusted negative binomial generalised estimating equation ^a^. | | | | | | | | | | | | | | | | | | |
| --- | --- | --- | --- | --- | --- | --- | --- | --- | --- | --- | --- | --- | --- | --- | --- | --- | --- | --- |
|  | Deprivation quintile | | | | | | | | | | | | | | |  | | |
|  | 1 (least deprived) | | | 2 |  |  | 3 |  |  | 4 |  |  | 5 (most deprived) | | | England | | |
|  | IRR | 95% CI | | IRR | 95% CI | | IRR | 95% CI | | IRR | 95% CI | | IRR | 95% CI | | IRR | 95% CI | |
| **Month** |  |  | |  |  | |  |  | |  |  | |  |  | |  |  | |
| Nov 2019 | ref | ref | ref | ref | ref | ref | ref | ref | ref | ref | ref | ref | ref | ref | ref | ref | ref | ref |
| Jun 2020 | 0.57 | 0.55 | 0.59 | 0.58 | 0.56 | 0.60 | 0.66 | 0.64 | 0.68 | 0.74 | 0.71 | 0.77 | 0.84 | 0.81 | 0.87 | 0.67 | 0.66 | 0.68 |
| Jul 2020 | 0.55 | 0.54 | 0.57 | 0.55 | 0.53 | 0.57 | 0.62 | 0.60 | 0.64 | 0.61 | 0.58 | 0.63 | 0.73 | 0.70 | 0.75 | 0.60 | 0.59 | 0.61 |
| Aug 2020 | 0.61 | 0.59 | 0.63 | 0.61 | 0.59 | 0.63 | 0.69 | 0.67 | 0.71 | 0.77 | 0.74 | 0.80 | 0.87 | 0.84 | 0.90 | 0.70 | 0.69 | 0.71 |
| Sept 2020 | 0.63 | 0.61 | 0.65 | 0.64 | 0.62 | 0.66 | 0.75 | 0.73 | 0.77 | 0.82 | 0.79 | 0.85 | 0.90 | 0.87 | 0.93 | 0.73 | 0.72 | 0.75 |
| Oct 2020 | 0.65 | 0.64 | 0.67 | 0.67 | 0.65 | 0.69 | 0.78 | 0.76 | 0.81 | 0.86 | 0.83 | 0.89 | 0.93 | 0.91 | 0.96 | 0.77 | 0.75 | 0.78 |
| Nov 2020 | 0.86 | 0.83 | 0.88 | 0.82 | 0.80 | 0.85 | 0.93 | 0.90 | 0.96 | 0.97 | 0.93 | 1.01 | 0.97 | 0.94 | 1.00 | 0.91 | 0.90 | 0.92 |
| Dec 2020 | 0.69 | 0.68 | 0.71 | 0.70 | 0.68 | 0.73 | 0.83 | 0.80 | 0.85 | 0.91 | 0.87 | 0.94 | 0.97 | 0.94 | 1.00 | 0.81 | 0.79 | 0.82 |
| Jan 2021 | 0.85 | 0.83 | 0.87 | 0.83 | 0.81 | 0.86 | 0.97 | 0.95 | 1.00 | 0.99 | 0.96 | 1.03 | 1.01 | 0.98 | 1.04 | 0.93 | 0.91 | 0.94 |
| Feb 2021 | 0.88 | 0.85 | 0.90 | 0.86 | 0.84 | 0.89 | 1.01 | 0.98 | 1.04 | 1.04 | 1.00 | 1.08 | 1.06 | 1.03 | 1.10 | 0.96 | 0.95 | 0.98 |
| Mar 2021 | 0.79 | 0.77 | 0.81 | 0.79 | 0.76 | 0.82 | 0.93 | 0.90 | 0.95 | 1.02 | 0.98 | 1.06 | 1.10 | 1.06 | 1.13 | 0.91 | 0.90 | 0.92 |
| Apr 2021 | 0.80 | 0.78 | 0.82 | 0.81 | 0.78 | 0.83 | 0.95 | 0.92 | 0.97 | 1.03 | 1.00 | 1.07 | 1.12 | 1.08 | 1.15 | 0.92 | 0.91 | 0.94 |
| May 2021 | 0.81 | 0.79 | 0.83 | 0.81 | 0.79 | 0.84 | 0.95 | 0.92 | 0.98 | 1.05 | 1.01 | 1.09 | 1.12 | 1.09 | 1.16 | 0.93 | 0.92 | 0.95 |
| Jun 2021 | 0.80 | 0.78 | 0.83 | 0.80 | 0.78 | 0.83 | 0.94 | 0.91 | 0.97 | 1.02 | 0.99 | 1.06 | 1.11 | 1.08 | 1.15 | 0.92 | 0.91 | 0.93 |
| Jul 2021 | 0.80 | 0.78 | 0.82 | 0.80 | 0.78 | 0.83 | 0.94 | 0.91 | 0.96 | 1.03 | 0.99 | 1.06 | 1.11 | 1.08 | 1.15 | 0.92 | 0.91 | 0.93 |
| Aug 2021 | 0.80 | 0.78 | 0.82 | 0.79 | 0.77 | 0.82 | 0.92 | 0.90 | 0.95 | 1.02 | 0.98 | 1.06 | 1.10 | 1.07 | 1.14 | 0.91 | 0.90 | 0.93 |
| Sept 2021 | 0.81 | 0.79 | 0.83 | 0.81 | 0.78 | 0.84 | 0.96 | 0.93 | 0.98 | 1.04 | 1.00 | 1.08 | 1.12 | 1.09 | 1.16 | 0.93 | 0.92 | 0.95 |
| Oct 2021 | 0.81 | 0.79 | 0.83 | 0.81 | 0.78 | 0.83 | 0.95 | 0.93 | 0.98 | 1.04 | 1.00 | 1.08 | 1.12 | 1.09 | 1.16 | 0.93 | 0.92 | 0.94 |
| Nov 2021 | 0.81 | 0.79 | 0.83 | 0.81 | 0.78 | 0.84 | 0.96 | 0.93 | 0.99 | 1.04 | 1.00 | 1.08 | 1.13 | 1.09 | 1.16 | 0.93 | 0.92 | 0.95 |
| Dec 2021 | 0.80 | 0.78 | 0.82 | 0.80 | 0.77 | 0.83 | 0.95 | 0.92 | 0.98 | 1.03 | 0.99 | 1.07 | 1.11 | 1.08 | 1.15 | 0.92 | 0.91 | 0.94 |
| Jan 2022 | 0.80 | 0.78 | 0.82 | 0.80 | 0.78 | 0.83 | 0.95 | 0.93 | 0.98 | 1.03 | 1.00 | 1.07 | 1.12 | 1.08 | 1.15 | 0.93 | 0.91 | 0.94 |
| Feb 2022 | 0.80 | 0.78 | 0.82 | 0.80 | 0.77 | 0.82 | 0.95 | 0.92 | 0.98 | 1.02 | 0.98 | 1.06 | 1.10 | 1.06 | 1.13 | 0.92 | 0.90 | 0.93 |
| Mar 2022 | 0.81 | 0.79 | 0.83 | 0.80 | 0.78 | 0.83 | 0.95 | 0.92 | 0.98 | 1.03 | 0.99 | 1.07 | 1.10 | 1.07 | 1.13 | 0.92 | 0.91 | 0.93 |
| Note: ^a^ adjusted for population density, rural urban classification and the number of food outlets in the physical food environment. | | | | | | | | | | | | | | | | | | |

| Supplementary Table 13: coefficients (coef.) and 95% confidence intervals (CI) for the percentage of food outlets registered to accept orders online in postcode districts in England, stratified by deprivation, calculated using an unadjusted generalised estimating equation ^a^. | | | | | | | | | | | | | | | | | | |
| --- | --- | --- | --- | --- | --- | --- | --- | --- | --- | --- | --- | --- | --- | --- | --- | --- | --- | --- |
|  | Deprivation quintile | | | | | | | | | | | | | | |  | | |
|  | 1 (least deprived) | | | 2 |  |  | 3 |  |  | 4 |  |  | 5 (most deprived) | | | England | | |
|  | coef. | 95% CI | | coef. | 95% CI | | coef. | 95% CI | | coef. | 95% CI | | coef. | 95% CI | | coef. | 95% CI | |
| **Month** |  |  | |  |  | |  |  | |  |  | |  |  | |  |  | |
| Nov 2019 | ref | ref | ref | ref | ref | ref | ref | ref | ref | ref | ref | ref | ref | ref | ref | ref | ref | ref |
| Jun 2020 | 0.47 | 0.43 | 0.51 | 0.54 | 0.50 | 0.58 | 0.54 | 0.51 | 0.58 | 0.64 | 0.60 | 0.69 | 0.83 | 0.79 | 0.88 | 0.60 | 0.58 | 0.62 |
| Jul 2020 | 0.45 | 0.41 | 0.50 | 0.52 | 0.47 | 0.57 | 0.48 | 0.43 | 0.53 | 0.52 | 0.45 | 0.58 | 0.69 | 0.62 | 0.76 | 0.53 | 0.50 | 0.55 |
| Aug 2020 | 0.56 | 0.52 | 0.60 | 0.63 | 0.57 | 0.68 | 0.61 | 0.56 | 0.66 | 0.69 | 0.63 | 0.75 | 0.92 | 0.86 | 0.97 | 0.67 | 0.65 | 0.69 |
| Sept 2020 | 0.28 | 0.24 | 0.32 | 0.30 | 0.26 | 0.33 | 0.26 | 0.22 | 0.29 | 0.25 | 0.22 | 0.29 | 0.23 | 0.20 | 0.26 | 0.25 | 0.23 | 0.26 |
| Oct 2020 | 0.34 | 0.31 | 0.38 | 0.35 | 0.31 | 0.39 | 0.31 | 0.27 | 0.35 | 0.30 | 0.26 | 0.33 | 0.28 | 0.25 | 0.32 | 0.30 | 0.29 | 0.32 |
| Nov 2020 | 0.51 | 0.46 | 0.55 | 0.51 | 0.46 | 0.56 | 0.45 | 0.41 | 0.49 | 0.39 | 0.35 | 0.43 | 0.36 | 0.33 | 0.40 | 0.42 | 0.40 | 0.44 |
| Dec 2020 | 0.39 | 0.35 | 0.44 | 0.40 | 0.35 | 0.44 | 0.35 | 0.32 | 0.39 | 0.33 | 0.29 | 0.38 | 0.31 | 0.28 | 0.35 | 0.34 | 0.32 | 0.36 |
| Jan 2021 | 0.54 | 0.48 | 0.59 | 0.53 | 0.48 | 0.58 | 0.47 | 0.42 | 0.51 | 0.42 | 0.38 | 0.47 | 0.40 | 0.36 | 0.44 | 0.44 | 0.42 | 0.46 |
| Feb 2021 | 0.60 | 0.55 | 0.65 | 0.59 | 0.54 | 0.64 | 0.53 | 0.49 | 0.57 | 0.49 | 0.45 | 0.54 | 0.47 | 0.43 | 0.50 | 0.51 | 0.49 | 0.53 |
| Mar 2021 | 0.56 | 0.52 | 0.61 | 0.57 | 0.52 | 0.61 | 0.50 | 0.46 | 0.55 | 0.48 | 0.43 | 0.53 | 0.47 | 0.43 | 0.51 | 0.49 | 0.47 | 0.51 |
| Apr 2021 | 0.58 | 0.53 | 0.62 | 0.57 | 0.52 | 0.62 | 0.52 | 0.48 | 0.57 | 0.51 | 0.46 | 0.56 | 0.50 | 0.46 | 0.54 | 0.51 | 0.49 | 0.53 |
| May 2021 | 0.59 | 0.54 | 0.63 | 0.59 | 0.54 | 0.64 | 0.54 | 0.49 | 0.58 | 0.53 | 0.48 | 0.58 | 0.52 | 0.48 | 0.56 | 0.53 | 0.51 | 0.55 |
| Jun 2021 | 0.58 | 0.54 | 0.63 | 0.58 | 0.54 | 0.63 | 0.54 | 0.50 | 0.58 | 0.53 | 0.48 | 0.58 | 0.52 | 0.48 | 0.56 | 0.53 | 0.51 | 0.55 |
| Jul 2021 | 0.59 | 0.54 | 0.63 | 0.59 | 0.54 | 0.63 | 0.54 | 0.50 | 0.59 | 0.54 | 0.49 | 0.58 | 0.53 | 0.49 | 0.57 | 0.53 | 0.51 | 0.55 |
| Aug 2021 | 0.61 | 0.56 | 0.66 | 0.60 | 0.55 | 0.64 | 0.55 | 0.50 | 0.59 | 0.56 | 0.51 | 0.61 | 0.53 | 0.49 | 0.58 | 0.54 | 0.52 | 0.56 |
| Sept 2021 | 0.62 | 0.57 | 0.66 | 0.59 | 0.55 | 0.64 | 0.55 | 0.51 | 0.60 | 0.56 | 0.51 | 0.62 | 0.54 | 0.49 | 0.58 | 0.55 | 0.53 | 0.57 |
| Oct 2021 | 0.62 | 0.57 | 0.67 | 0.61 | 0.56 | 0.65 | 0.57 | 0.52 | 0.62 | 0.58 | 0.53 | 0.63 | 0.55 | 0.51 | 0.59 | 0.56 | 0.54 | 0.58 |
| Nov 2021 | 0.63 | 0.59 | 0.68 | 0.62 | 0.57 | 0.66 | 0.58 | 0.53 | 0.63 | 0.58 | 0.53 | 0.63 | 0.58 | 0.53 | 0.62 | 0.57 | 0.55 | 0.59 |
| Dec 2021 | 0.62 | 0.57 | 0.67 | 0.60 | 0.55 | 0.65 | 0.58 | 0.53 | 0.62 | 0.57 | 0.52 | 0.62 | 0.56 | 0.52 | 0.61 | 0.56 | 0.54 | 0.58 |
| Jan 2022 | 0.63 | 0.58 | 0.68 | 0.61 | 0.57 | 0.66 | 0.59 | 0.54 | 0.63 | 0.57 | 0.52 | 0.62 | 0.57 | 0.53 | 0.62 | 0.57 | 0.55 | 0.59 |
| Feb 2022 | 0.63 | 0.59 | 0.68 | 0.62 | 0.58 | 0.67 | 0.59 | 0.54 | 0.64 | 0.58 | 0.53 | 0.63 | 0.56 | 0.51 | 0.60 | 0.57 | 0.55 | 0.59 |
| Mar 2022 | 0.65 | 0.60 | 0.70 | 0.63 | 0.58 | 0.68 | 0.60 | 0.55 | 0.65 | 0.58 | 0.53 | 0.63 | 0.56 | 0.52 | 0.61 | 0.58 | 0.56 | 0.60 |
| ^a^ percent calculated as the number of food outlets registered to accept orders online compared to number of food outlets in the physical food environment. | | | | | | | | | | | | | | | | | | |

| Supplementary Table 14: coefficients (coef.) and 95% confidence intervals (CI) for the percentage of food outlets registered to accept orders online in postcode districts in England, stratified by deprivation, calculated using an adjusted generalised estimating equation ^a^. | | | | | | | | | | | | | | | | | | |
| --- | --- | --- | --- | --- | --- | --- | --- | --- | --- | --- | --- | --- | --- | --- | --- | --- | --- | --- |
|  | Deprivation quintile | | | | | | | | | | | | | | |  | | |
|  | 1 (least deprived) | | | 2 |  |  | 3 |  |  | 4 |  |  | 5 (most deprived) | | | England | | |
|  | coef. | 95% CI | | coef. | 95% CI | | coef. | 95% CI | | coef. | 95% CI | | coef. | 95% CI | | coef. | 95% CI | |
| **Month** |  |  | |  |  | |  |  | |  |  | |  |  | |  |  | |
| Nov 2019 | ref | ref | ref | ref | ref | ref | ref | ref | ref | ref | ref | ref | ref | ref | ref | ref | ref | ref |
| Jun 2020 | 0.50 | 0.46 | 0.54 | 0.59 | 0.54 | 0.63 | 0.59 | 0.55 | 0.64 | 0.72 | 0.67 | 0.78 | 0.85 | 0.81 | 0.90 | 0.67 | 0.64 | 0.69 |
| Jul 2020 | 0.48 | 0.44 | 0.53 | 0.57 | 0.51 | 0.62 | 0.53 | 0.47 | 0.59 | 0.58 | 0.50 | 0.66 | 0.71 | 0.64 | 0.78 | 0.58 | 0.55 | 0.61 |
| Aug 2020 | 0.60 | 0.55 | 0.64 | 0.68 | 0.62 | 0.74 | 0.67 | 0.61 | 0.72 | 0.77 | 0.70 | 0.85 | 0.94 | 0.88 | 1.00 | 0.74 | 0.72 | 0.77 |
| Sept 2020 | 0.30 | 0.26 | 0.34 | 0.32 | 0.28 | 0.36 | 0.28 | 0.24 | 0.31 | 0.28 | 0.24 | 0.32 | 0.23 | 0.20 | 0.27 | 0.27 | 0.26 | 0.29 |
| Oct 2020 | 0.37 | 0.33 | 0.41 | 0.38 | 0.34 | 0.43 | 0.33 | 0.29 | 0.37 | 0.33 | 0.29 | 0.37 | 0.29 | 0.26 | 0.32 | 0.33 | 0.31 | 0.35 |
| Nov 2020 | 0.54 | 0.49 | 0.59 | 0.56 | 0.50 | 0.61 | 0.48 | 0.44 | 0.53 | 0.43 | 0.39 | 0.48 | 0.37 | 0.33 | 0.40 | 0.46 | 0.44 | 0.48 |
| Dec 2020 | 0.42 | 0.37 | 0.46 | 0.43 | 0.38 | 0.49 | 0.38 | 0.34 | 0.43 | 0.37 | 0.32 | 0.42 | 0.32 | 0.28 | 0.36 | 0.37 | 0.35 | 0.39 |
| Jan 2021 | 0.57 | 0.52 | 0.62 | 0.58 | 0.52 | 0.63 | 0.50 | 0.45 | 0.55 | 0.47 | 0.42 | 0.53 | 0.41 | 0.37 | 0.44 | 0.49 | 0.47 | 0.51 |
| Feb 2021 | 0.63 | 0.58 | 0.69 | 0.65 | 0.60 | 0.71 | 0.58 | 0.53 | 0.62 | 0.55 | 0.50 | 0.60 | 0.48 | 0.44 | 0.51 | 0.56 | 0.54 | 0.58 |
| Mar 2021 | 0.60 | 0.55 | 0.65 | 0.62 | 0.57 | 0.67 | 0.55 | 0.50 | 0.59 | 0.54 | 0.48 | 0.59 | 0.48 | 0.44 | 0.52 | 0.54 | 0.52 | 0.57 |
| Apr 2021 | 0.61 | 0.56 | 0.66 | 0.63 | 0.57 | 0.68 | 0.57 | 0.52 | 0.62 | 0.57 | 0.51 | 0.63 | 0.51 | 0.46 | 0.55 | 0.56 | 0.54 | 0.58 |
| May 2021 | 0.62 | 0.57 | 0.67 | 0.65 | 0.60 | 0.70 | 0.58 | 0.53 | 0.63 | 0.59 | 0.53 | 0.65 | 0.53 | 0.49 | 0.57 | 0.58 | 0.56 | 0.60 |
| Jun 2021 | 0.62 | 0.57 | 0.66 | 0.64 | 0.59 | 0.69 | 0.59 | 0.54 | 0.63 | 0.59 | 0.54 | 0.65 | 0.53 | 0.49 | 0.58 | 0.58 | 0.56 | 0.60 |
| Jul 2021 | 0.62 | 0.57 | 0.67 | 0.64 | 0.59 | 0.70 | 0.59 | 0.54 | 0.64 | 0.60 | 0.54 | 0.66 | 0.54 | 0.49 | 0.58 | 0.59 | 0.56 | 0.61 |
| Aug 2021 | 0.64 | 0.59 | 0.69 | 0.65 | 0.60 | 0.70 | 0.60 | 0.54 | 0.65 | 0.62 | 0.57 | 0.68 | 0.54 | 0.50 | 0.59 | 0.60 | 0.58 | 0.62 |
| Sept 2021 | 0.65 | 0.60 | 0.70 | 0.65 | 0.60 | 0.70 | 0.60 | 0.55 | 0.65 | 0.63 | 0.57 | 0.69 | 0.55 | 0.50 | 0.59 | 0.60 | 0.58 | 0.63 |
| Oct 2021 | 0.66 | 0.61 | 0.71 | 0.66 | 0.61 | 0.72 | 0.62 | 0.57 | 0.67 | 0.64 | 0.59 | 0.70 | 0.56 | 0.52 | 0.60 | 0.62 | 0.59 | 0.64 |
| Nov 2021 | 0.67 | 0.62 | 0.72 | 0.67 | 0.62 | 0.73 | 0.63 | 0.58 | 0.68 | 0.65 | 0.59 | 0.71 | 0.59 | 0.54 | 0.63 | 0.63 | 0.61 | 0.65 |
| Dec 2021 | 0.66 | 0.61 | 0.71 | 0.66 | 0.60 | 0.71 | 0.63 | 0.57 | 0.68 | 0.63 | 0.57 | 0.70 | 0.57 | 0.53 | 0.62 | 0.62 | 0.59 | 0.64 |
| Jan 2022 | 0.67 | 0.62 | 0.72 | 0.66 | 0.61 | 0.71 | 0.64 | 0.59 | 0.69 | 0.64 | 0.58 | 0.70 | 0.58 | 0.54 | 0.63 | 0.63 | 0.60 | 0.65 |
| Feb 2022 | 0.67 | 0.62 | 0.72 | 0.68 | 0.63 | 0.73 | 0.64 | 0.59 | 0.70 | 0.65 | 0.59 | 0.71 | 0.57 | 0.52 | 0.61 | 0.63 | 0.60 | 0.65 |
| Mar 2022 | 0.69 | 0.64 | 0.74 | 0.69 | 0.63 | 0.74 | 0.66 | 0.60 | 0.71 | 0.65 | 0.59 | 0.71 | 0.57 | 0.52 | 0.62 | 0.64 | 0.61 | 0.66 |
| Note: ^a^ percentage calculated as the number of food outlets registered to accept orders online compared to number of food outlets in the physical food environment. Model adjusted for population density and rural urban classification. | | | | | | | | | | | | | | | | | | |

| Supplementary Table 15: Operationalisation of matching monthly and quarterly data ^a^. | |
| --- | --- |
| **Month** | **Quarter** |
| November 2019 | Q4 2019 |
| June 2020 | Q3 2020 |
| July 2020 | Q3 2020 |
| August 2020 | Q3 2020 |
| September 2020 | Q4 2020 |
| October 2020 | Q4 2020 |
| November 2020 | Q4 2020 |
| December 2020 | Q1 2021 |
| January 2021 | Q1 2021 |
| February 2021 | Q1 2021 |
| March 2021 | Q2 2021 |
| April 2021 | Q2 2021 |
| May 2021 | Q2 2021 |
| June 2021 | Q3 2021 |
| July 2021 | Q3 2021 |
| August 2021 | Q3 2021 |
| September 2021 | Q4 2021 |
| October 2021 | Q4 2021 |
| November 2021 | Q4 2021 |
| December 2021 | Q1 2022 |
| January 2022 | Q1 2022 |
| February 2022 | Q1 2022 |
| March 2022 | Q1 2022 |
| Notes: ^a^ Monthly data from the online food delivery service. ^b^ Quarterly data from Ordnance Survey’s Points of Interest dataset. | |
